# Environmental Sustainability and Physicochemical Property Screening of Chitin and Chitin-Glucan from 22 Fungal Species

**DOI:** 10.1021/acssuschemeng.4c01260

**Published:** 2024-05-09

**Authors:** Vanessa Grifoll, Paula Bravo, Maria Nieves Pérez, Margarita Pérez-Clavijo, Marta García-Castrillo, Aitor Larrañaga, Erlantz Lizundia

**Affiliations:** † Mushroom Technological Research Center of La Rioja (CTICH), Ctra. Calahorra km 4, Autol 26560, La Rioja, Spain; ‡ BCMaterials, Basque Center for Materials, Applications and Nanostructures, Edif. Martina Casiano, Pl. 3 Parque Científico UPV/EHU Barrio Sarriena, Leioa 48940, Biscay, Spain; § SGIker, General Research Services, University of the Basque Country (UPV/EHU), Barrio Sarriena, Leioa 48940, Biscay, Spain; ∥ Life Cycle Thinking Group, Department of Graphic Design and Engineering Projects. University of the Basque Country (UPV/EHU), Plaza Ingeniero Torres Quevedo 1, Bilbao 48013, Biscay, Spain

**Keywords:** chitin, fungi, renewable carbon, glucans, biorefinery, bioproducts, valorization

## Abstract

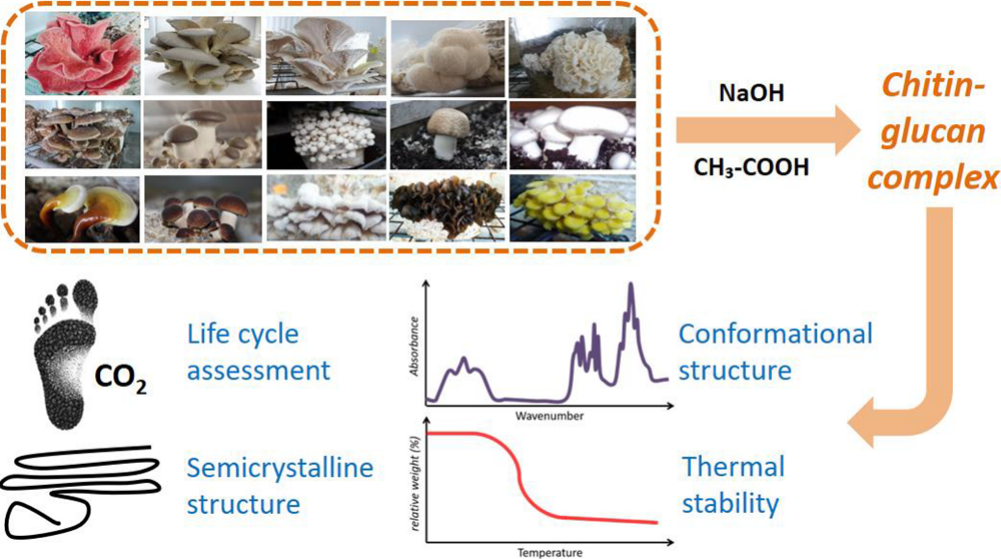

Thanks to its biobased character with embedded biogenic carbon, chitin can aid in the transition to a sustainable circular economy by replacing fossil carbon from the geosphere. However, meeting current demands for material availability and environmental sustainability requires alternative methods limiting conventional chemical and energy-consuming chitin extraction from crustaceans. To assist future chitinous bioproduct development, this work analyzes the physicochemical properties and potential environmental sustainability of fungal chitin-glucan complexes. A conventional isolation procedure using sodium hydroxide, a weak acid, and short reaction times are applied to the fruiting body of 22 fungal species. Besides, the valorization of underutilized waste streams including *Agaricus bisporus* and *Agaricus brunnescens* stipes is investigated. The carbohydrate analysis renders chitin fractions in the range of 9.5–63.5 wt %, while yields vary from 4.2 to 29.9%, and the *N*-acetylation degree in found in between 53.0 and 98.7%. The sustainability of the process is analyzed using life cycle assessment (LCA), providing impact quantification for global warming potential, terrestrial acidification, freshwater eutrophication, and water use. With 87.5–589.3 kg·CO_2_-equiv per kilo, potentially lower global warming potential values in comparison to crustacean chitin are achieved. The crystallinity degree ranged from 28 to 78%, while the apparent chitin crystalline size (*L*_020_) is between 2.3 and 5.4 nm. Ten of the species yield α-chitin coexisting with semicrystalline glucans. Zwitterionic properties are observed in aqueous solutions, shifting from cationic to anionic at pH 4.5. With its renewable carbon content, fungal chitin is an environmentally sustainable alternative for high-value applications due to its balance of minimal treatment, low carbon footprint, material renewability, ease of isolation, thermal stability, zwitterionic behavior, biodegradability, and noncytotoxicity.

## Abstract 2

Chitin and chitin-glucan complexes are isolated from 22 fungal species to study their semicrystalline structure, conformational features, thermal stability, and environmental sustainability.

## 1 Introduction

The transition from a linear economy reliant on fossil-derived materials to a sustainable circular economy necessitates the development of transformative and renewable materials with low carbon emissions.^1,2^ To achieve this, the exploitation of renewable carbon feedstocks from underutilized biological resources can effectively address current global challenges associated with the depletion of primary raw materials, and accumulation of nonbiodegradable products in terrestrial, river, or marine ecosystems.^3^ biobased materials such as cellulose, chitin, or lignin have been specially attractive of being composed of biogenic carbon. In other words, the utilization of these materials does not result in the release and subsequent delivery of fossil carbon from the geosphere into the atmosphere. Instead, this renewable carbon can circulate between the biosphere in the form of biomass, the atmosphere, or the technosphere as technical materials, creating a carbon circular economy.^4^ As such, biobased materials are being the subject of intense research efforts regarding their isolation, functionalization, and potential applications.^5−7^ Overall, natural polymers fulfill the often self-excluding requisites of low economic cost, local availability, ease of processing, competitive thermo-mechanical performance, and (bio)degradability.^8^ Their unique multidimensional structure has been exploited to develop multiple high-tech uses, including photonic colored materials,^9^ electrolytes for safer batteries with extended operation lifespan,^10^ shape memory materials, and inks for additive manufacturing.^11^

Although chitin has not been explored as extensively as cellulose, it has great potential for developing multifunctional materials that meet current environmental sustainability demands. However, without the implementation of economically and environmentally feasible isolation processes, the potential of chitin-containing biomass to meet today’s global challenges can be hardly realized. The biomass heterogeneity, especially in the case of crustacean exoeskeletons, is another bottleneck toward high-tech applications, where a careful control over the resulting molecular structure needs to be taken.^12^ The compositional variability of chitin sources could be addressed under controlled cultivation conditions of biological entities that can produce chitin.

Interestingly, chitin is naturally present in many living organisms^7^ and has additional benefits over other biopolymers. Some of these advantages include its significant structural strength and flexibility,^9^ its hierarchical structure,^5^ its piezoelectricity, or its use as a starting material for chitosan,^7^ a cationic polysaccharide with multiple applications.^5^ Despite its low allergenicity, biocompatibility, and biodegradability, the current uses of chitin remain elusive due to the complexity of its isolation. Considering that over 10^5^ mmt (million metric ton) of chitin are annually produced in the aquatic biosphere,^4^ the primary source of chitin is currently found in the exoskeleton of arthropods, with shrimp shells being particularly prevalent. Conventional chitin isolation process involve a top-down demineralization (typically 1–4 M HCl) to remove calcium carbonate from the shells,^13^ followed by deproteinization under alkaline conditions. The generation of abundant wastewater during chitin isolation and the use of mineral acids poses a challenge to the environmentally sustainable use of chitin.^13^ Therefore, new strategies with improved environmental and economic prospects should be considered for the full exploitation of chitin in advanced material applications.

Several works have highlighted the potential of nonconventional chitin sources for increased sustainability and tailored multifunctionality.^7,14,15^ Fungi are particularly relevant in this context due to their ease of harvesting, lack of seasonal and regional variation, and the vast number of species that remain to be explored. In 2022, the global mushroom market was estimated to be worth USD 54.9 billion. Such already large market value is projected to expand at a compound annual growth rate of 9.7% from 2022 to 2030 largely due to the increasing vegan population.^16^ Production quantities of the main mushrooms are for shiitake mushrooms (*Lentinula edodes*), followed by oyster mushrooms (*Pleurotus* spp.), wood-ear mushrooms (Auricularia spp.), and button mushrooms (*Agaricus bisporus*).^17^ This production is greatly driven by the demand for edible mushrooms (with Asia-Pacific region accounting for 78.6% of the global revenue), together with the implementation of modern cultivation techniques.^16^ Additionally, the nutritional and health benefits of this food are becoming more widely known. Fungal cell walls are composed mainly of chitin, glucan, and proteins,^18^ where chitin contents ranges from 0.5 to 40.0 wt % (dry mass) depending on the species.^19^ This value is slightly above the 15.0–20.0 wt % chitin found in hard crustacean shells.^20^ Fungal chitin can be extracted easily, in an environmentally friendly and cost-effective manner, without the need for a demineralization step that relies on strong inorganic acids such as HCl. This is achieved by alkaline deproteinization and mechanical blending at lower temperatures and shorter times when compared to the protocols used to isolate chitin from crustacean exoeskeletons.^21−23^ In addition, the alkali treatment prevents excessive deacetylation and chain degradation.^24^ Importantly, life cycle assessment results have shown a significantly lower carbon footprint for nanochitin isolated from white mushrooms compared to nanochitin isolated from shrimp shells (18.5 and 906.8 kg CO_2_-equiv·kg^–1^, respectively).^13^ This is due to increased extraction yields, reduced energy consumption due to shorter and lower temperature reactions, and reduced chemical requirements. Therefore, fungi are expected to provide a more sustainable source of chitin compared to current marine-based processes. As there are many fungal species that have not been fully explored as potential chitin sources, we hypothesize that a comprehensive screening of the environmental sustainability of chitin obtained from different fungal species would provide valuable information for future sustainable bioproduct development.

The properties of biobased polymers, such as crystallinity, physicochemical conformation, and thermal stability, depend on the source of the raw material and the isolation process.^7,25,26^ Chitin isolation has been investigated in common white mushrooms (*Agaricus bisporus*)^21−23^ and bracket fungi (*Daedaleopsis confragosa*).^27^ Controlling the properties and ensuring reliable production remains a challenge in implementing chitin into real-world applications. To unlock the potential of fungal-derived chin,^4^ it is important to study how the chitin source (fungal species) affects the resulting material. Besides, the economic viability of chitin isolation may vary from species to species due to the fact that the growth of fungal species is largely limited by water and soil availability, and the final yield may strongly correlate to the type of fungi. Therefore, this work investigates the structural, physicochemical, and thermal properties of chitin isolated from the complete fruiting body of 22 fungal species. The study focuses on the most widely consumed species, including the *Agaricus* genus (*A. bisporus*, *A. brunnecens*), *Pleurotus* genus (*P. ostreatus*, *P. eryngii*), and *Lentinula edodes*. Other species have also been included due to their nutritional and health benefits, such as the *Pholiota, Flammulina, Hericium, Grifola, Agrocybe, Calocybe, Hypsizigus, Ganoderma* and other species of the *Pleurotus* genus (*P. salmoneo*, *P. citrinopilleatus*, and *P. ferulae*). In addition, the stipes of *Agaricus bisporus* and *Agaricus brunnescens* were also utilized due to their potential to generate underutilized waste streams. The ultimate goal is not to optimize the chitin extraction process from mushrooms using different reagents, but to screen the physicochemical properties and environmental sustainability of different mushroom species as chitin sources.

## 2 Materials and Methods

### 2.1 Fungal Sources

The following mushrooms were cultivated in the Technological Mushroom Centre (CTICH—La Rioja, Spain): *Agaricus bisporus* (AB), *Agaricus bisporus* var. *brunnescens* (ABp), *Agaricus bisporus* var. *subrufescens (ABz)*, *Auricularia Auricularia-judae (AAj)*, *Agrocybe aegerita* (AA), *Calocybe indica* (CI), *Flammulina veluptites* var. yellow (FVa), *Flammulina velutipes* var. white (FVb), *Ganoderma lucidum* (GAN), *Grifola frondose* (GF), *Hericium coralloides* (HC), *Hericium erinaceus* (HE), *Hypsizigus tessulatus* var. white (HTb), *Hypsizigus tessulatus* var. gray (HTg), *Lentinula edodes* (LE), *Pholiota nameko* (PN), *Pleurotus eryngii* (PE), *Pleurotus ostreatus* (PO), *Pleurotus citrinopileatus* (PC), *Pleurotus ferulae* (PF), *Pleurotus salmoneo* (PS), and *Schizophyllum commune* (SC). Freeze-dried mushroom flours were generated using whole mushrooms. The mushrooms were harvested based on their maturity stage, rather than their cap size. The optimal maturity of the mushroom is determined by a round cap, a completely intact veil, and a long enough stem for trimming. Mushrooms grown on both pasteurized (AB, ABp, ABz) and sterilized substrate (the rest) were selected.

### 2.2 Reagents

Sodium hydroxide (NaOH) pellets (99%), sodium carbonate anhydrous (Na_2_CO_3_, 99.5–100.5%), ammonium hydroxide solution (NH_4_OH, 28–30% w/w), glacial acetic acid (CH_3_COOH, > 99.7%), ethanol (C_2_H_6_O, > 99.8%), and hydrochloric acid (HCl, 37%) were purchased from Panreac (Barcelona, Spain). Acetylacetone, p-dimethylaminobenzaldehyde (DMAB), and HPLC-grade glucosamine, glucose, xylose, mannose, and galactose standards were purchased from Sigma-Aldrich (Steinheim, Germany). All reagents were used in highly pure grades. Demineralized water was used to prepare alkaline and acidic aqueous solutions.

### 2.3 Chitin Isolation

Chitin isolation was carried out according to the protocol described by Hassainia et al.^28^ First, fresh samples were cleaned and subsequently freeze-dried (LyoQuest, Telstar) at −54 °C for 72 h to avoid undesired enzymatic degradation. After grinding using a Retsch ZM200 blender (Haan, Germany), 4 g of powdered mushrooms were treated in 1 M NaOH at 80 °C for 120 min at a ratio 1:30 (w/v) under continuous stirring for deproteinization. After that, the solution was centrifuged at 5000 rpm during 10 min, and the supernatant was discarded. The alkali insoluble residue was subjected to washing/centrifugation cycles with demineralized water several times until neutral pH. Finally, the alkali insoluble residue was freeze-dried at −54 °C for 72 h. Chitin was isolated upon stirring the freeze-dried alkali insoluble residue in acetic acid (2% v/v) at a ratio of (1:100 w/v) at 95 °C for 6 h. The insoluble material was recovered by centrifugation at 5000 rpm during 10 min, washed several times with demineralized water until pH became neutral, and finally freeze-dried. The result is the isolated chitin. Note that the study utilized low solid:liquid ratios to follow a general procedure for chitin isolation so the yield, composition, and physicochemical properties of the resulting chitin from a variety of fungal sources could be screened. These ratios have room for optimization.

### 2.4 Glucosamine Determination

The chitin concentration is represented by glucosamine, so its determination was performed according to Son et al. with modifications.^29^ In the first place, the freeze-dried chitin isolate was subjected to acid hydrolysis. Here, 40 mg of sample was hydrolyzed with 20 mL of 6 M HCl at a ratio 1:2 (w/v) at 100 °C for 16 h. The solution was neutralized with 6 M NaOH and centrifuged at 500 rpm during 10 min. Afterward, 1 mL of the neutralized hydrolysate was mixed with 1 mL of Acetylacetone solution (Acetylacetone in 0.5 M Na_2_CO_3_, 1:50 (v/v)) at 95 °C for 10 min. Next, 1 mL of DMAB solution (0.8 g of DMAB in 30 mL of ethanol and 30 mL of HCl solution) was added to the solution, and it was reacted at 75 °C for 30 min. After, the solution was cooled in ice, and absolute ethanol was added to adjust the volume to 10 mL. Lastly, the absorbance of the final solution was measured at 530 nm using a spectrophotometer (Multiskan multiplate, Thermo Fisher). The complete reaction procedure, excluding the hydrolysis, was performed using a d-glucosamine standard, and its standard curve was used to calculate the total glucosamine content in the chitin isolates.

### 2.5 Carbohydrate Analysis

The freeze-dried powdered samples from 22 mushroom species were analyzed to determine their total carbohydrate content. The phenol-sulfuric method based on Dubois et al. with modifications was used for the analysis.^30^ Prior hydrolysis of the samples was necessary. Each sample (50 mg) was hydrolyzed with 2.5 mL of 2.5 M HCl at 100 °C for 3 h. After cooling to room temperature, the volume was adjusted to 50 mL with miliQ water and neutralized with sodium carbonate. The hydrolysates were centrifuged at 5000 rpm for 10 min and then diluted as required. Next, 1 mL of the sample was mixed with 1 mL of 5% phenol and 5 mL of 98% sulfuric acid in a tube. After 10 min, the mixture was shaken well and placed in a water bath at 25 °C for 20 min before measuring the absorbance at 490 nm using a Multiskan GO Microplate Spectrophotometer (Thermo Fisher). Glucose content was used as the standard to calculate the percentage of carbohydrates in each mushroom sample.

### 2.6 Life Cycle Assessment

The environmental impacts of isolating chitin and chitin-glucan from fungi have been quantified using the life cycle assessment (LCA) methodology. To facilitate the transition from laboratory-scale research to practical implementation, a simulated bench-scale process treating 1 kg of fungi was studied while maintaining the lab-scale stoichiometry. The *cradle-to-gate* environmental impacts were determined based on the International Standards ISO 14040/14044. This includes the raw material acquisition, chemical and energy needs for *on-site* isolation, and required waste treatments. The process flowchart is provided in Figure S1 for future comparison. The electricity mix of the Spanish grid during November 2023 was utilized, which comprises a renewable share of 62.8% (the full distribution is shown in Table S1). The assessment utilized OpenLCA software with the ecoinvent v3.9 database, which integrates the IPCC 2021 guidelines. The impacts of global warming potential, terrestrial acidification, and freshwater eutrophication were determined using the ReCiPe 2016 Midpoint (H) method due to its wide consensus. Besides, the AWARE (Available WAter REmaining) method development was applied to account for water use (wastewater treatment was excluded in this case). The functional unit (FU) for this study is 1 kg of dried isolated material (so the results are normalized to the amount of isolated material), as it represents the most relevant underlying physical relationship.^31^

### 2.7 Structural and Physicochemical Characterization

Room temperature X-ray diffraction (XRD) of freeze-dried chitin powder was conducted in a PHILIPS X’PERT PRO automatic diffractometer in theta–theta configuration, secondary monochromator with Cu–Kα radiation (λ = 1.5418 Å), and a PIXcel solid state detector (active length in 2θ 3.347°). Data were collected from 5 to 80° 2θ (step size = 0.026 and time per step = 400 s) scan speed 0.0167° s^–1^, at RT. A variable divergence slit giving a constant 7 mm area of sample illumination was used. Attenuated total reflectance Fourier transform infrared (ATR-FTIR) spectra of freeze-dried chitin powder were obtained using a Jasco FT/IR-6100 spectrometer (ATR optics; 2 cm^–1^ resolution). Zeta-potential of aqueous chitin dispersions for pH values ranging from 2 to 11 was obtained using a Malvern Zetasizer Nano-ZS. The pH was tuned upon addition of 0.05 M NaOH or 0.05 M HCl. The thermodegradation of freeze-dried chitin powder (5–10 mg samples) was assessed in a TA Instruments TGA model Q50-0545 in platinum pans. Samples were heated from room temperature to 600 °C at a heating rate of 10 °C min^–1^ with a 60 mL min^–1^ N_2_ flow.

### 2.8 Statistical Analysis

Experimental results are expressed as means ± standard error of the mean. Variance one-way analysis (ANOVA) followed by Tukey’s test were used to distinguish differences between means at *p* < 0.05. Statgraphics Centurion 19 software (Version 19.6.03, Statgraphics Technologies Inc.) was used for that purpose.

## 3 Results and Discussion

### 3.1 Extract Yield, Composition, and Environmental Footprint

**Figure 1:**
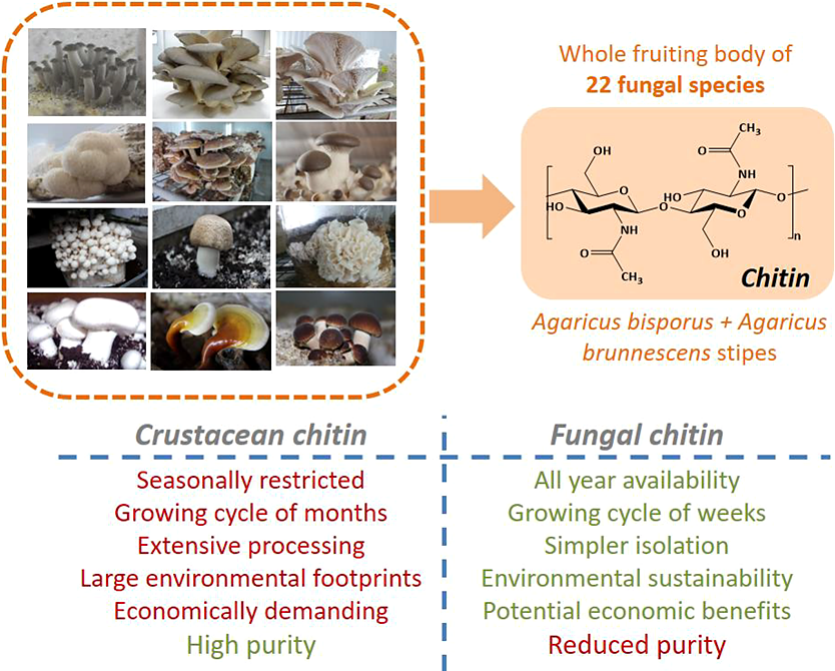
Outline of the study focusing on the isolation of chitin and chitin-glucan complex from 22 fungal species. The key attributes of fungal chitin compared to crustacean chitin are summarized.

The aim of this study is to isolate chitin and chitin-glucan complexes from the fruiting bodies of 22 fungal species and to analyze the environmental sustainability, structural, physicochemical, and thermal properties of the resulting material. Additionally, the feasibility of using stipes from commonly cultivated fungi as a source of chitin was evaluated. Figure 1 provides a summary of the top-down approach that was employed. The fungi were washed, freeze-dried, treated with an aqueous alkaline solution to remove proteins, lipids, and glucans, neutralized, treated again with acetic acid, washed, and finally freeze-dried to obtain a powder-like material. The applied process does not apply mechanical blending for isolation in contrast with the process reported by Nawawi et al.,^21^ who obtained nanofibrillar chitin. Besides, the isolation process was carried out with special care to minimize any unwanted deacetylation of the chitin, so no decolorization step was included. Chitin isolation from fungi offers several significant advantages over its crustacean counterpart. Fungi are available all year round and have an easy-to-grow/fast-grow cycle compared to the conventional chitin source (crustacean shells). Moreover, recent studies have shown that the chitin isolation procedures from fungi show improved environmental^13^ and economic sustainability,^32^ with a comparatively safer character (in terms of cytotoxicity and inflammatory effects) than related biobased nanomaterials.^33^ However, these advantages come at the expense of material purity.

The yield of chitin isolation on a dry weight basis is summarized in Table 1-left for all studied fungal species. Values ranging from 4.3 ± 0.2 to 29.9 ± 0.5 wt % were obtained. The yield observed for *Agaricus bisporus* is comparable to that previously reported (6.4–7.4 wt %).^28,34^ A correlation was observed between yield and mushroom systematics, such as species, where two distinguished groups have been identified. An average yield of 18.7% has been observed for the species *Ganoderma lucidum* (29.9 ± 0.6 wt %), *Lentinula edodes* (17.9 ± 0.4 wt %.), *Auricularia judae* (16.3 ± 0.3 wt %), *Agrocybe aegerita* (14.7 ± 0.5 wt %), and *Flammulina velutipes* var. yellow, respectively, which are particularly attractive for the establishment of a prospective fungal biorefinery for the commercialization of chitin.^32^ Other species have a significantly reduced average yield of 8.7%. Additionally, significant differences were observed between the white and yellow varieties of Hypsizigus tessulatus. Similarly, significant differences were detected among Pleurotus species, particularly in the case of *P. ostreatus* (4.43 ± 1.05 wt %). Importantly, obtained yields clearly demonstrate the potential of multiple species for chitin valorization that have been underestimated so far. It is worth noting that the fungal stipes of *Agaricus bisporus* and *Agaricus brunnescens* allow higher yields compared to the whole fruiting body (10.11–10.60 wt % vs the 7.54 and 8.35 wt % obtained for the whole fungi). Additionally, literature reports suggest that small variations in the chitin extraction protocol, such as temperature, time, or NaOH concentration, are required for each mushroom species to achieve maximum yield in chitin isolation. For instance, the chitin yield for *Pleurotus ostreatus*, *Flammulina velutipes*, and *Lentinula edodes* can be improved up to 20.9 wt % by using hot water reflux for 30 min at 85 °C before alkali treatment.^24^

**Table 1:**
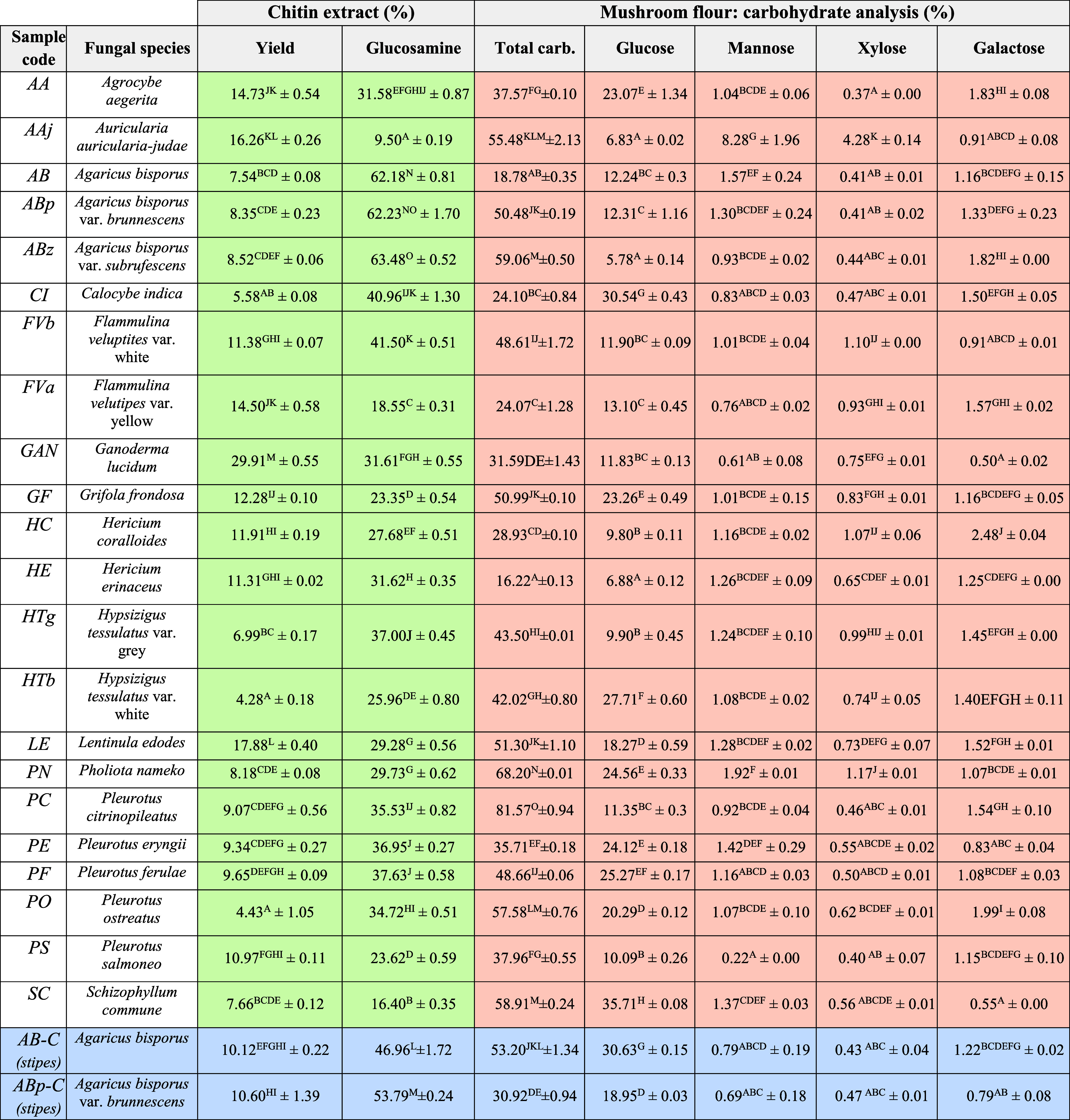
Yield of Isolated Material and Glucosamine from Chitin Extract (Highlighted in Green), Carbohydrate Analysis from Freeze-Dried Mushrooms from the Whole Fruiting Body of 22 Fungal Species (Highlighted in Orange)^a^

a The stipes for *Agaricus bisporus* and *Agaricus brunnescens* were also included (highlighted in blue). Different letters denote statistical differences between species according to Tukey’s HSD (honestly significant difference) test. Games-Howell post hoc test has been used to Glucosamine analysis (variances are different).

The carbohydrate content in freeze-dried samples was also determined, and the results are summarized in Table 1-right. Carbohydrate is a major component in mushrooms and its total content typically is found between 35 and 70% dry weight depending on the species.^35^ In this context, the *P. citrinopileatus* species showed the highest carbohydrate content (81.57 ± 0.94%), while the *A. bisporus* has the lowest content (18.78 ± 0.35%). In this sense, glucose is the major saccharide component (5.78 ± 0.14 to 35.71 ± 0.08%) in all the species except for *A. auricula-judae*, in which the majority saccharide is mannose (8.28 ± 1.96%). A similar mannose content (10.7%) for *A. auricula-judae* has been reported by Kadnikova et al.,^37^ although a larger glucose concentration was observed. Such differences may originate from the cultivation procedure and seasonal variations. Finally, low concentrations of galactose and xylose have been detected (0.50 ± 0.02 to 2.48 ± 0.04%; 0.37 ± 0.00 to 4.28 ± 0.14%, respectively). The sugar content in mushrooms, including xylose, mannose, and maltose, has been found to be low, and no correlation between sugar content and mushroom systematics, such as species or varieties, is observed. The chitin concentration, represented by glucosamine, remains in the range of 9.5–63.5 wt % for the studied fungal sources. Of all the species studied, the genus *Agaricus* (*bisporus, brunnescens*, and *subrufescens*) has the highest glucosamine content. The large variation in chitin content depends on different specific strategies among fungal species to fulfill the structural support function in the cell wall.^36^ According to Vetter, chitin content appears to be a characteristic of a species and independent of its varieties.^34^

It should be noted that not all nonchitinous content can be removed during the alkaline extraction process, given the occurrence of covalent cross-links between chitin and glucans.^38^ This characteristic differs significantly from lignocellulosic resources, where cellulose, lignin, and hemicellulose are physically bound and could be fractionated to yield nearly pure compounds.^39^ In this context, glucans are major structural polysaccharides of the fungal cell wall, constituting approximately 50–60 wt % of the wall, and a fraction of these glucans are alkali insoluble, so they will be present in the chitin extract. These results highlight an increased purity of chitin isolated by the process employed here when compared to chitin extracted by simple alkali treatment at 65 °C and mechanical blending, which yields chitin/glucan ratios of 50/50 and 35/65 for *Agaricus bisporus* cap and stalk, respectively (note that mechanical blending resulted into a nanofibrillar material).^21^

**Figure 2:**
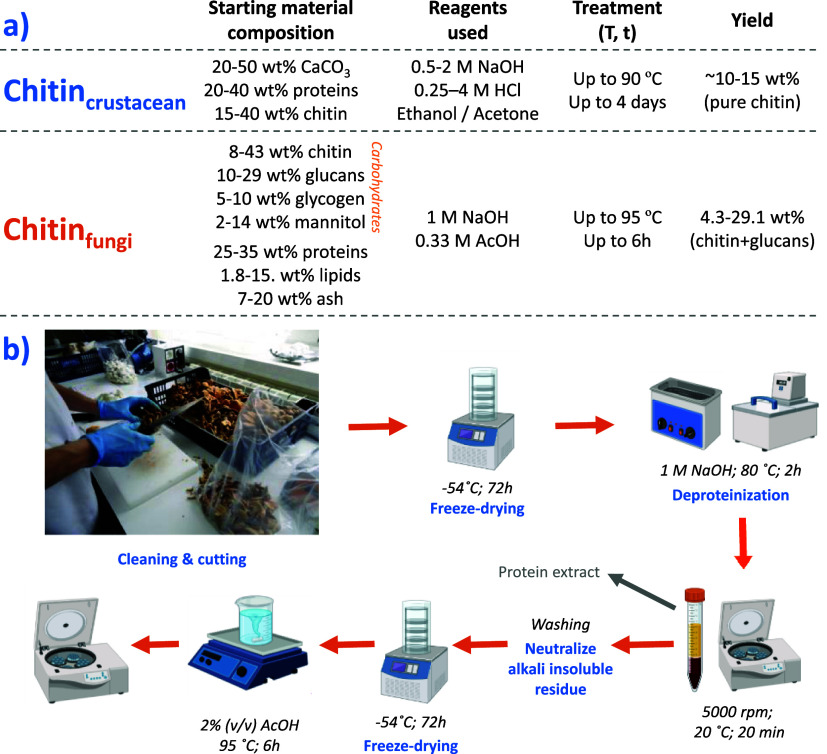
(a) Main characteristic of chitin isolation from crustacean shells and fungi; (b) process definition of the chitin isolation followed in this work.

**Figure 3:**
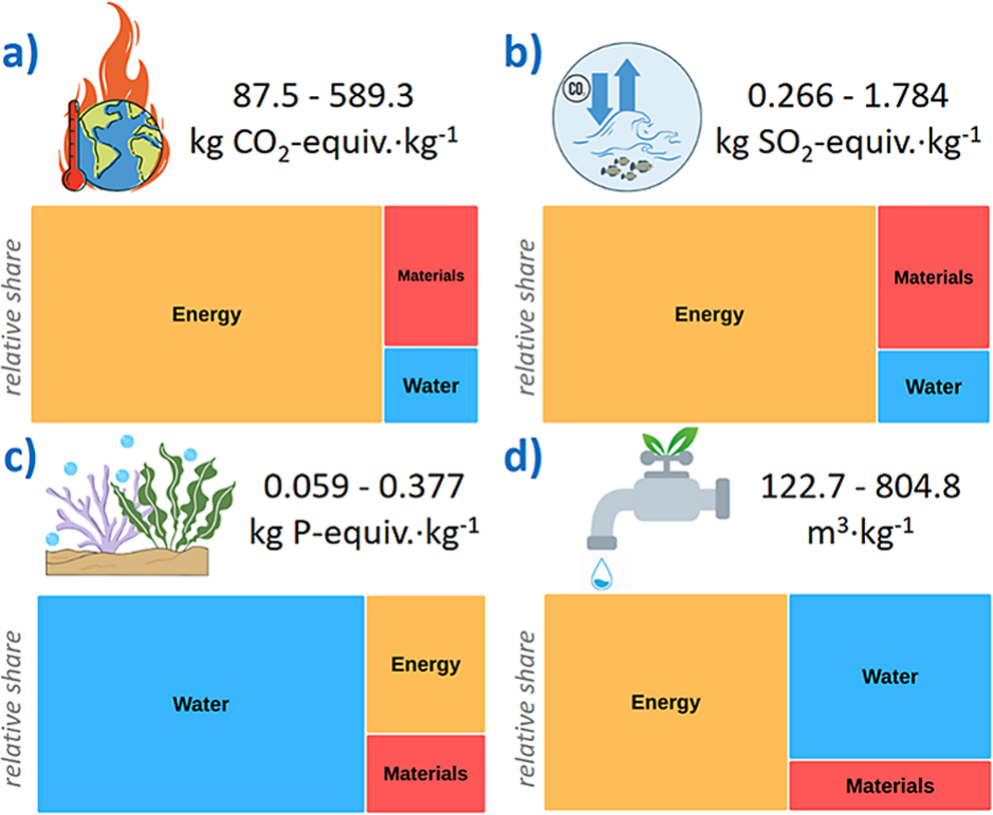
Environmental impacts for the categories of (a) global warming potential; (b) terrestrial acidification; (c) freshwater eutrophication; and (d) AWARE water use. The relative contribution of energy consumption, materials utilization, and water consumption are provided for each impact category.

Figure 2a,b schematically summarizes the approximate process characteristics for the isolation of chitin from crustacean shells and fungi. Note that this data represents approximate values given the wide variety of biomass available for chitin valorization. Importantly, in spite of the multicomponent composition of fungi, the chitin isolation from this resource can be accomplished with a reduced amount of chemicals (lower concentrations and no ethanol or acetone for washing), using a weak acid such as acetic acid in comparison with the conventionally utilized strong HCl, potential reactions (which is translated into reduced energy consumption) at higher yields. To verify that such a procedure is translated into chitin and chitin-glucan materials having reduced environmental impacts, we conducted a life cycle assessment (LCA) to quantify the cradle-to-gate environmental impacts. The global warming potential (GPW, quantified in equivalent CO_2_ emissions) impact category is first presented due to its crucial role in assessing the environmental impact of materials and processes. Note that no credits were accounted for carbon capture in the biomass, which would further lower the impacts. Therefore, only the bench-scale process for isolation is considered. The LCA results are summarized in Figure 3, while Table 2 provides the environmental impacts for each of the species (see Table S2 for further information). Results indicate that chitin and chitin-glucan could be isolated from fungi with a CO_2_ footprint of 87.5–589.3 kg CO_2_-equiv. kg^–1^. Such footprint differences mostly originate from the yield and small differences in the quantity of AcOH required for chitin isolation. These results highlight the potential of fungal chitin-glucan to potentially compete chitin isolation from shrimp shells, whose greenhouse emissions account for 47 kg CO_2_-equiv. kg^–1^ when considering a large-scale facility producing 50 ton chitosan annually^40^ or the 677 kg CO_2_-equiv. kg^–1^ reported for a bench-scale chitin isolation from 1 kg of shrimp shell waste.^13^ In this context, the avoidance of harsh acid hydrolysis treatment for demineralization is a key advantage. Additionally, higher yields can be attained compared to chitin isolated from crustacean sources (9.7% extract yield for Cancer pagurus crab,^21^ or 3.0% yield for shrimp shells).^40^ The relatively straightforward isolation procedure when compared to chitin isolation from crustacean exoskeletons also provides additional environmental benefits.^41^ However, it should be noted that the carbon footprint remains prohibitively high to effectively support the production of sustainable bioproducts; especially, considering that the GWP of wood-based staple fiber production, for example, 1.2-to-2.5 kg CO_2_-equiv. kg^–1^.^4^ Potential GWP reductions could be achieved by increasing the currently poor solid:liquid ratios (1:30 for mushroom:NaOH and 1:100 for chitin:AcOH) to ensure improved atom efficiency of the process and reduce water utilization and waste generation.

**Table 2:**
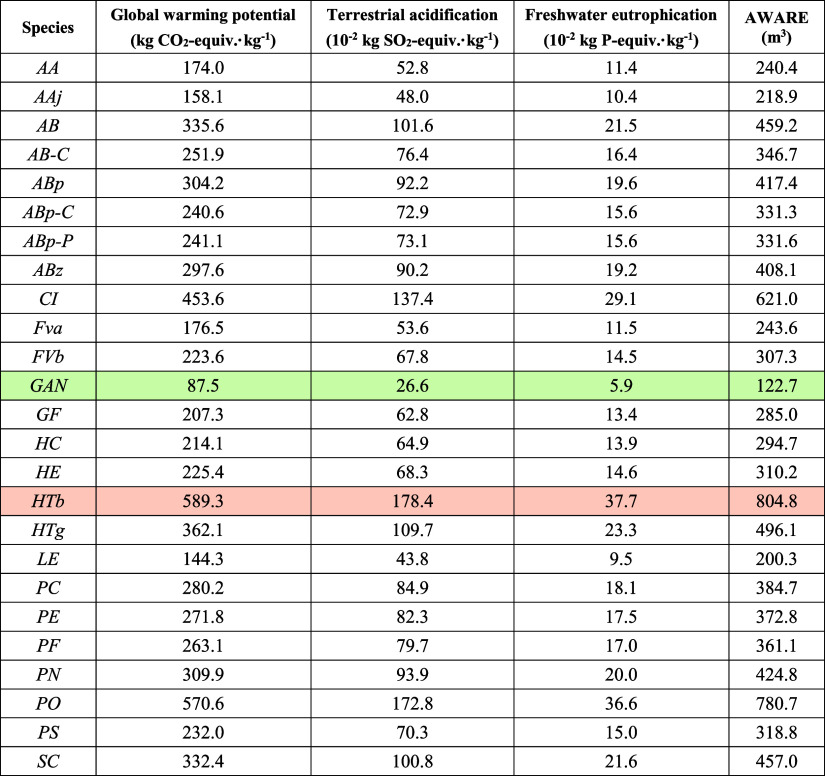
Environmental Impacts of Chitin and Chitin-glucan Isolation from 22 Fungal Species in the Categories of Global Warming Potential, Acidification, Eutrophication, and Water Use^a^

a The lowest and highest impacts are highlighted in green and yellow, respectively.

For other categories, values ranging from 0.266 to 1.784 kg SO_2_-equiv. kg^–1^ are obtained for terrestrial acidification, 0.059 to 0.377 kg N-equiv. kg^–1^ for freshwater eutrophication, and 122.7–804 m^3^ kg^–1^ for water use according to the AWARE method. These results are lower than the impacts reported for chitin derivatives such as chitin nanocrystals from crab shells (2.054 kg SO_2_-equiv. kg^–1^ and 0.494 kg N-equiv. kg^–1^),^13^ which demonstrates the potential sustainability of fungi for chitin valorization. In addition, it can be observed that electricity used for isolation has the largest share on the impact category of GWP, reaching a relative share of 77.8–79.6% depending on the species. The energy use also accounts for the 72.5–75.6% of the terrestrial acidification impact and contributes to 54.6% on average of the water use due to the hydropower needs. In this sense, a combination of up-scaling and a shift to a fully renewable electric power supply could significantly reduce the impacts in all the categories (estimates indicate reductions of up to 6.5 times upon up-scaling).^42,43^ Importantly, AcOH, NaOH and the water should be recycled in the near term upon collection and subsequent separation of the wastewater to reduce the overall environmental impact. This is particularly relevant for the impact category of freshwater eutrophication, where water and material use share an 83% contribution.

### 3.2 Semicrystalline Structure and Conformational Features

**Figure 4:**
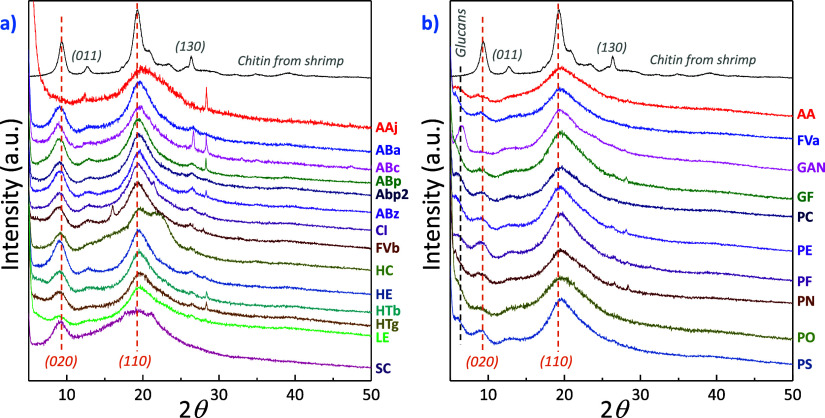
XRD patterns of freeze-dried samples: (a) samples containing chitin and (b) samples containing chitin and semicrystalline glucans.

The semicrystalline and conformational characteristics of the isolated material has been investigated to explore the potential uses of fungal chitin. Figure 4 displays the X-ray diffraction (XRD) patterns of the freeze-dried isolated material and the pattern corresponding to commercial chitin powder extracted from shrimp shells. The material obtained exhibits a combination of crystalline and amorphous regions, with two main diffraction peaks centered at 2θ ∼ 8.0° and ∼19.0° originating from the (020) and (110) planes of α-chitin (2-chain orthorhombic unit cell with chains organized in an antiparallel arrangement), respectively.^44,45^ Weaker peaks located at 2θ ∼ 12.0° and 26° assigned to the (011) and (130) crystal planes, respectively, can be observed in certain samples. Considering also the glucosamine analysis shown above, the extracts in Figure 4a are composed of α-chitin and noncrystalline organic matter. However, it should be noted that in 10 of the analyzed samples (Figure 4b), the presence of semicrystalline glucan (β-glucans) is detected at 2θ ∼ 6.0°.^46^ This material is a group of β-d-glucose polysaccharides with a linear backbone containing 1–3 β-glycosidic linkages.^47^ Although the results indicate that the initial glucan content in fungi may influence the final structure of the obtained extract (*Agaricus bisporus* is a low glucan-content mushroom, and the obtained extract shows no trace of semicrystalline glucans), further studies are needed to explore this correlation. For instance, *Pleurotus ostreatus* contains a high glucan concentration, and its extract is composed of semicrystalline glucan.^47,48^ It should be noted that the presence of glucans does not necessarily imply a loss of physicomechanical properties. This is because the β-d-glucose polysaccharides actually enhance the mechanical flexibility, improve film-forming properties,^21,22^ and facilitate gel formation.^23^

The diffraction patterns suggest that the material isolated from fungi has a semicrystalline structure, as demonstrated by the crystalline Bragg reflections as well as a broad amorphous halo. Quantifying the crystallinity degree can facilitate comparisons of the structures of the material isolated from different fungal species and compare them with the one resulting from commercial chitin derived from shrimp shells. After the deconvolution of the 22 XRD patterns shown in Figure S2, we quantified the crystallinity degree of the samples according to^49^
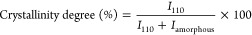
1where *I*_110_ and *I*_amorphous_ represents the intensity at 2θ ∼ 19.0° and of the amorphous halo at 2θ ∼ 21–23°, respectively. As summarized in Table S3, estimated crystallinity degrees vary greatly, ranging from 28 to 78%. In all cases, the crystallinity degree remains below the 76% reported for the pure shrimp shell chitin,^41^ the 85% for chitin nanocrystals isolated by HCl hydrolysis,^50^ or the 92% achieved for chitin nanocrystal isolated from waste shrimp shells by TEMPO-oxidation.^51^ This suggests that the isolation process used to extract the chitin (using a weak acid and short reaction times) preserves the low molecular amorphous chitin. Furthermore, the presence of amorphous glucans between semicrystalline α-chitin regions can lower the overall crystalline fraction.^21^ These results agree well with the literature, with observed crystallinity degrees of 48% for chitin extracted from *Hypsizygus marmoreus*,^52^ 50% for the chitin-glucan isolated from *Komagataella pastoris*,^46^ 62% for the chitin-glucan isolated from *Aspergillus niger*,^46^ 44–63% observed for α-chitin isolated from *Agaricus bisporus*,^28,53^ or 80% achieved for *Pleurotus eryngii*.^52^ The results suggest that fungi have a wide range of chitin-glucan ratios, as the same isolation process produces significantly different crystallinity indexes.^54,55^

To gain more insight into the semicrystalline structure of the isolated material, we calculated the apparent chitin crystalline size (*L*_020_) from the deconvoluted X-ray diffraction data using Scherrer′s equation:^56^
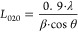
2where β is the width at half height of the peak related to (020) lattice plane, θ is the diffraction angle of the (020) reflection, and λ represents the incident wavelength. The crystalline size for the transversal axis of the orthorhombic geometry of α-chitin, as summarized in Table S3, remains in the range of 2.3–5.4 nm, which is comparable to *L*_020_ = 3.4–3.7 nm observed by Nawawi et al. for chitin (nanofibrils in this case) isolated from *Agaricus bisporus*.^21^ On average, a larger apparent *L*_*020*_ is seen in samples where α-chitin and semicrystalline glucans coexist. As such, the smaller *L*_020_ is observed for the *AAj* species (2.3 nm, solely α-chitin), while a *L*_020_ of 5.4 nm is detected for *GF*, *PC*, *PE*, and *PF* species (α-chitin and glucans). It is important to note that these *L*_020_ values are significantly lower than 8–9 nm observed for chitin nanocrystals from crustacean shells.^50^ This smaller crystalline size is consistent with the reduced crystallinity degree observed and is a characteristic of poorly crystalline biopolymers.^57^

**Figure 5:**
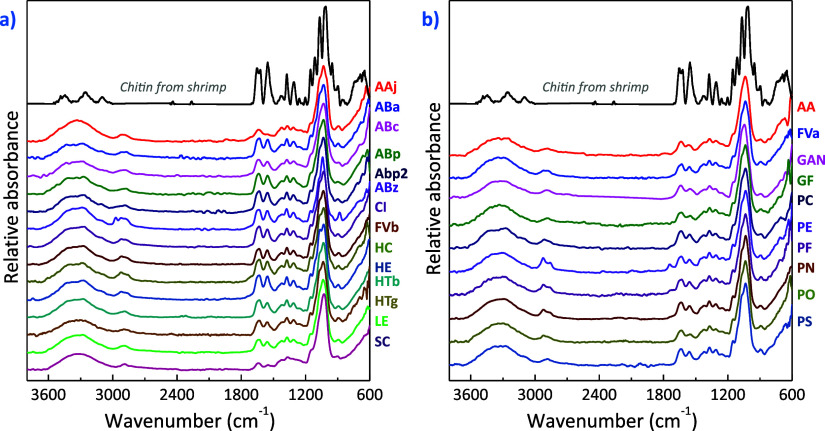
FTIR spectra of freeze-dried samples: (a) samples containing pure chitin and (b) samples containing chitin and semicrystalline glucans.

Fourier transform infrared spectroscopy (FTIR) is a suitable method for studying conformation and packing mode of chitin macromolecules. Chitin chains interact through H-bonding between amine and carbonyl groups in different ways depending on the crystalline polymorphs (α-, β-, and γ-chitin).^44^ The FTIR spectra of the extracts obtained are grouped into those containing pure chitin and those containing chitin-glucan in Figure 5a,b, respectively. Overall, the samples exhibit features that correspond to the typical absorption bands of chitin. Note that further details on band position for each fungal source are given in Table S4. In summary, all samples display a broad band in the 3650–3200 cm^–1^ region from the O–H stretching of hydroxyl groups at 3435–3333 cm^–1^ plus the – NH stretching at 3314–3250 cm^–1^ (peak position depends on the source). A narrower band at 2925–2880 cm^–1^ originating from the – CH groups is also observed.^23^ The characteristic amide I, amide II, and amide III bands can be seen 1640–1629, 1564–1552, and 1322–1309 cm^–1^, respectively.^22^ Additionally, a small stretching peak at 1424–1414 cm^–1^ originates from the C–N of the indole structure in melanin,^21^ which is responsible for the brownish color in some of the extracts. The signal for the CH bending and symmetric CH_3_ deformation appears at 1376–1366 cm^–1^. Besides, a sharp band at 1045–1028 cm^–1^ originating from the C–OH stretching attributable to chitin is observed.^22^ Finally, the β-glycosidic −CH deformation is observed at 901–887 cm^–1^.

The spectra for the extracts in Figure 5b show a decrease in absorbance of the amide peaks compared to the 1045–1028 cm^–1^ absorption band, indicating the presence of carbohydrate glucans, which is consistent with the XRD results. Furthermore, the weak peak at ∼1730 cm^–1^ can be attributed to the β-(1,6) linkage in glucan units present in the extracts.^46^ Importantly, no traces of glycosylated proteins (glycoproteins, proteins with oligosaccharide chains covalently attached to amino acid side-chains) were observed as the 1540 cm^–1^ stretching vibration for proteins was absent in all compositions.^58^ This is explained by the fact that the glycoproteins are removed from the extract through a process of dissolution upon alkali treatment, protein extraction, washing, and acetic acid treatment.^28^

The presence of secondary amides is indicated by the III bands (centered at 1320 cm^–1^) observed in all the extracts, confirming that the isolated material is composed of chitin rather than chitosan. Therefore, our isolation process, which utilizes 1 M NaOH at 80 °C for 120 min, is able to maintain the native structure of chitin. To confirm the extent of the transformation of *N*-acetyl-d-glucosamine to d-glucosamine, we obtained the degree of *N*-acetylation (DA) of chitin. This was measured from the specific band appearing at 1655 cm^–1^ of the amide I band as a measure of the *N*-acetyl group content. Additionally, A_3430_ was used as an internal standard, which corresponds to the absorbance at 3430 cm^–1^ due to hydroxyl group as^59,60^
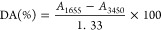
3where 1.33 represents the ratio of this absorbance for a fully acetylated compound. It is important to note that this method offers a quick approach to estimate the degree of *N*-acetylation, where the results offer a similar accuracy as those obtained by ^1^H NMR.^59,60^Table S5 shows that the obtained chitin extract had DA values ranging from 53.0% for the *GF* species, to 98.7% for the *ABp-c* species, indicating that all the extracts were composed of chitin rather than chitosan. Specifically, 26% of the extracts had a DA ≥ 80%, 48% had DA values from 60 to 80%, and the remaining extracts had DA values from 50 to 60%. For comparison purposes, DA values of 63.4–69.8% have been reported for *Agaricus bisporus* depending on the body part,^28^ while raw α-chitin DA is around 94% (DA obtained using FTIR spectroscopy in both cases).^61^ These results demonstrate the effectiveness of the proposed method for extracting chitin with minimal *N*-deacetylation.

### 3.3 ζ-Potential

**Figure 6:**
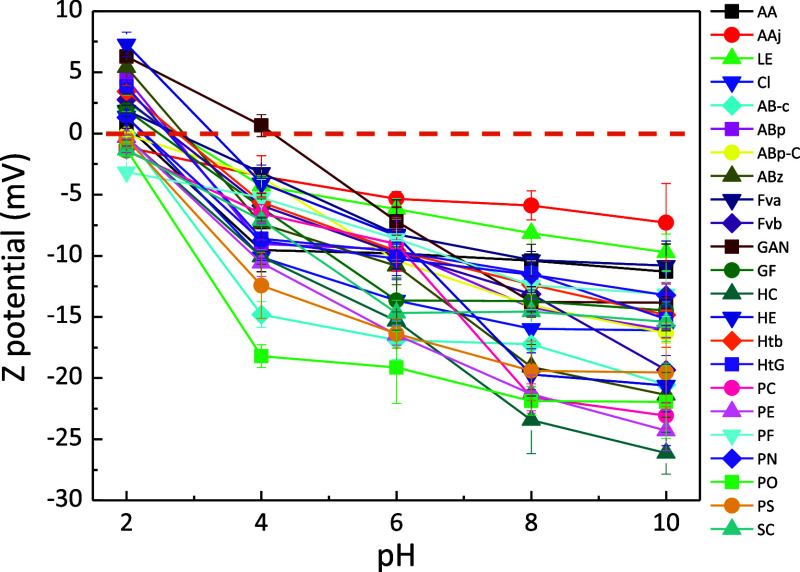
ζ-Potential of chitin aqueous dispersions (0.05 wt %) at pH values from 2 to 10. The 0 mV value is highlighted by an orange dashed line.

The electrostatic interaction of a given material with their surrounding environments largely defines its application in fields such as energy storage or active packaging. For example, a positive correlation is seen between ζ-potential and antibacterial activity in polysaccharides (the higher the ζ-potential, the higher the activity against *Escherichia coli* and *Staphylococcus aureus*).^62^ The ζ-potential of isolated extracts was measured as a function of pH, and the results are summarized in Figure 6. Further details are given in Figure S3. In all the extracts, the ζ-potential decreased as the pH increased from 2 to 10. In summary, the amino groups in chitin become highly protonated at low pH values (acidic environment), resulting in a positive surface charge of approximately +3 mV on average at pH 2.0. As the pH increases, the ζ-potential decreases to reach values from −7 to −26 mV at pH 10, depending on the fungal source. This is due to the continuous deprotonation of chitin′s dissociable groups as the media becomes more basic. Changes in ζ-potential may be attributed to variations in the amount of surface-exposed *N*-acetyl groups and chitin purity.^63^ Interestingly, the samples with lower ζ-potential at pH = 2 exhibit a plateau in ζ-potential when the pH remains above 6. This effect could be due to charge compensation between the amino groups of chitin and the negatively charged residuals (glucans and other polysaccharides), resulting in lower surface charges under acidic conditions.^22^

The isoelectric point (IEP), the pH value at which the surface charge becomes zero (see the dashed line), is observed at a pH of 2-to-4.1. However, in certain samples, a negative ζ-potential is observed over the studied pH range. The IEP values achieved remain closely related to the values observed for pure α-chitin (IEP of 3.5)^64^ or the IEP = 2.7 reported for chitin (nanofibrils in this case) isolated from *Agaricus bisporus*,^23^ where the presence of amorphous glucans lowers the overall surface charge at a given pH value. For comparison, the IEP of pure chitosan is found at a pH value of 9,^63^ demonstrating that the extracted products are predominantly composed of chitin. Overall, the zwitterionic characteristic of chitin extracts bearing both positive and negative chemical groups is interesting for developing fungal-derived materials for Pickering emulsions^65^ or water remediation.^66^

### 3.4 Thermal Stability

**Figure 7:**
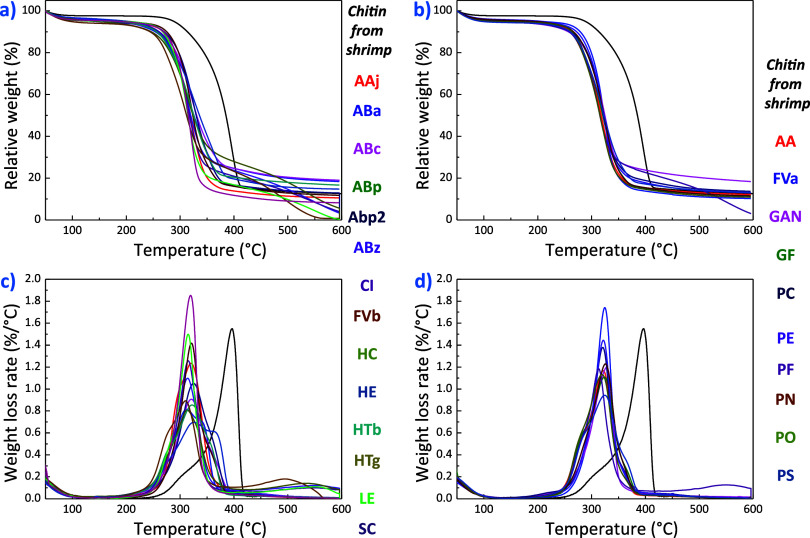
Thermogravimetric curves of freeze-dried samples: (a) containing pure chitin and (b) containing chitin and semicrystalline glucans. Weight loss rates of freeze-dried samples: (c) containing pure chitin and (d) containing chitin and semicrystalline glucans.

The thermal stability of chitin is crucial for its potential practical applications. Improved thermal stability means better resistance to irreversible changes in the chemical and physical structure when exposed to higher temperatures.^67^ As such, we assessed the thermal stability of fungal chitin using thermogravimetry. Figure 7 shows the thermogravimetric analysis (TGA) curves of fungal chitin obtained at a heating rate of 10 °C min^–1^ under N_2_ atmosphere. Additionally, the respective characteristic thermodegradation temperatures (the temperatures at which the first 10 wt % loss and the maximum degradation rate are achieved, defined as *T*_10%_ and *T*_peak_, respectively) and maximum degradation rates (α_max_) are summarized in Table S6. The samples exhibit comparable thermodegradation processes, with chitin displaying its three characteristic decomposition steps.^68^ An initial loss of approximately 5–10 wt % is observed at 80 °C, which is due to the evaporation of the adsorbed water. Subsequently, a significant thermodegradation event comprising a ∼60 wt % loss is observed in the 240–380 °C range. This event is caused by the depolymerization and decomposition of 2-amino-2-deoxy-d-glucopyranose units through a random breaking of C–O–C skeletal bonds,^68^ resulting in the release of light gaseous compounds such as NH_3_, H_2_O, CO, and CO_2_. Ultimately, above 400 °C, the pyranose ring degrades (15 wt % contribution). No evidence of thermodegradation steps at ∼180 and ∼250 °C can be observed, indicating a successful removal of proteins, lipids, and pigments.^28^ The char residue (containing nitrogen and aromatic structures) at 600 °C significantly varies between extracts, ranging from a minimum of 0.5–21 wt % (pure α-chitin has a char residue of 15 wt %).^69^ In the future, pyrolysis-gas chromatography/mass spectrometry studies will be needed to reveal the mechanism of thermodegradation due to the complexity of the process. It is important to note that the maximum thermodegradation rate of fungal chitin occurs at lower temperatures than commercial α-chitin from shrimp shells (396.5 °C), which matches literature observations.^46^ Despite having reduced thermal stability,^28^ fungal chitin is actually more thermally stable than β-chitin, which undergoes thermodegradation at 281–329 °C.^70^ This is due to a combination of factors, including a lower degree of acetylation, lower crystallinity degree, and the presence of polysaccharide residuals.

## 4 Conclusions

This study utilized a conventional chitin isolation procedure with a weak acid to investigate the potential of different fungal species as environmentally sustainable sources of chitin and chitin-glucan complexes. The fruiting bodies of 22 fungal species, including the edible *Agaricus bisporus* and its variations, *Agrocybe aegerita*, *Calocybe indica*, and *Flammulina velutipes*, were screened. This approach can increase yields compared to using the complete fruiting body of their particular species. Additionally, the isolation of chitin was performed utilizing a weak acid with short reaction times at relatively low temperature as opposed to the methods utilizing strong mineral acids, such as HCl. The global warming potential values ranged from 87.5 to 589.3 kg·CO_2_ equiv. per kg of material. These values are lower than the greenhouse gas emissions that result during chitin isolation from shrimp shells. The analysis of impact distribution suggests the need to implement a closed-loop recycling system with 100% renewable energy for environmentally friendlier isolation processes. The yield, *N*-acetylation degree, crystallinity degree, and crystallite size depend on the source of chitin, with values ranging from 4.3 to 29.9 wt %, 53.0–98.7, 28–78%, and 2.3–5.4 nm, respectively. Besides, the isolated material exhibits zwitterionic behavior, with positive charges at low pH values and negative charges when the pH exceeds 4.5. This behavior makes them a versatile and renewable platform material for environmental remediation purposes. Additionally, the chitin extract is stable at temperatures up to ∼240 °C, which is above the maximum processing temperature of many thermoplastics.

Furthermore, the stipes of mushrooms, which are usually discarded as low-value animal feed or compost, can be utilized to produce materials with comparable properties and larger extraction yield. This finding paves the way for integrated fungal biorefineries that can utilize the entire fungi. Considering their renewable carbon content, in combination with their balance of thermal stability, zwitterionic behavior, renewability, and potential biodegradability, along with the widespread cultivation of the studied species and the high fraction of waste generated during mushroom production, these fungi offer clear attributes to play a relevant role as economically and environmentally sustainable chitin feedstock. Accordingly, this work lays the foundation for expanding the practical implementation and impact of fungal chitin on global sustainable material challenges.

## Data Availability

All the data used to support the findings of this study are included within the article.

## Supporting Information Available

Fungi growth process, life cycle flowchart, energy mix distribution, environmental impact distribution, crystalline structure parameters, FTIR bands, degree of *N*-acetylation, thermodegradation temperatures, deconvoluted XRD patterns, and ζ-potential at varying pH values of chitin and chitin-glucan complexes are shown (PDF)
