# Supplementary material for: Environmental Sustainability and Physicochemical Property Screening of Chitin and Chitin-Glucan from 22 Fungal Species
Source: ACS Sustain Chem Eng. 2024 May 9;12(20):7869–81. doi: 10.1021/acssuschemeng.4c01260 (PMC11110056; doi:10.1021/acssuschemeng.4c01260)
Supplement: Supplementary file 1 — sc4c01260_si_003.pdf [file sc4c01260_si_003.pdf]

Supporting Information

# Environmental sustainability and physico-chemical property screening of chitin and chitin-glucan from 22 fungal species

*Vanessa Grifoll,[1] Paula Bravo,[1] Maria Nieves Pérez, [1] Margarita Pérez-Clavijo,[1] Marta García-Castrillo,[2] Aitor Larrañaga,[3] Erlantz Lizundia[2,4]**

[1] *Mushroom Technological Research Center of La Rioja (CTICH), Ctra. Calahorra km 4, 26560 Autol (La Rioja), Spain.*

[2] *BCMaterials, Basque Center for Materials, Applications and Nanostructures, Edif. Martina Casiano, Pl. 3 Parque Científico UPV/EHU Barrio Sarriena, 48940 Leioa, Biscay, Spain.*

[3] *SGIker, General Research Services, University of the Basque Country (UPV/EHU), Barrio Sarriena, 48940 Leioa, Biscay, Spain.*

[4] *Life Cycle Thinking Group, Department of Graphic Design and Engineering Projects. University of the Basque Country (UPV/EHU), Plaza Ingeniero Torres Quevedo 1, 48013 Bilbao, Biscay, Spain*

*: Corresponding author: erlantz.liizundia@ehu.eus

Number of pages: 16

Number of tables: 6

Number of figures: 3

## Material and methods

**Fungal Sources.** Mushrooms need specific growing conditions, in which the adequate substrate plays an important role. The substrate suitable for fungal growth and reproduction must provide the specific nutritional requirements for each fungal species and be free of or have a low level of possible competitor fungi and bacteria. The substrate is used by the mycelium for energy and nutrition. The mycelium is the network of hyphae. This fine network of cells at one stage of its life cycle, fructifies in the form of mushrooms.

Some mushrooms, such as *A.bisporus*, are able to develop selective substrates through composting (decomposition of organic matter and subsequent pasteurization). However, for most cultured bacterial species, selective substrates have not been developed and it is necessary to eliminate competitors by sterilizing the substrate. There are mushrooms that can grow in both pasteurized and sterile substrates, e.g. oyster mushroom (*Pleurotus ostreatus*), oyster mushroom (*Pleurotus eryngii*), shiitake (*Lentinus edodes*). Of the 22 species tested, only the *Algaricus* species (*A.bisporus, A.brunnecens* and *A.subrufencens*) were carried out with pasteurized substrate.

The most widespread method of producing substrate for mushroom and oyster mushroom cultivation is pasteurised compost. These substrates are made from lignocellulosic and proteinaceous materials, such as cereal straws and various manures, urea and water.

The process consists of three phases:

- Phase I or Fermentation: wetting of the raw materials, mixing and subsequent degradation under controlled conditions (8-9 days).
- Phase II or Pasteurization: disinfection of the compost to eliminate competitors (12-14 days). At the end of the composting process, inoculation is carried out with fungal mycelium.
- Phase III: The compost fully colonized by the mycelium after a period of incubation and ready to apply the casing material (14-18 days)

All phases are carried out in tunnels with an aerated floor and a specific regulation system. The result is a very homogeneous compost which, in the cultivation phase, gives a uniform and high yield. Casing material applied on top of the colonized mycelium required to obtain a profitable production, in which the morphological switch from the vegetative to the reproductive stage takes place. Fructification relies on this material. Stage of fructification is a transition from vegetative growth (mycelium) to reproductive growth (mushrooms).

The aim of the sterilized substrate is to eliminate practically all the organisms present on a substrate, whether they are spores, bacteria, etc. Four types of raw materials are used in sterilized substrate: sawdust, agricultural by-products, grain or supplements and other supplements. Sawdust and agricultural by-products are used in larger quantities. Sawdust is used to give structure to the mixture and to facilitate aeration. The second group of materials also provides food for the fungus. Both are known as base ingredients.

The most used sawdust comes from oak barrels used in wine cellars. It is an easily available raw material in our country, Spain; in cooperages and, moreover, it is wood without any type of residue or treatment. Therefore, oak sawdust is a perfect raw material for our purpose. As for the agricultural by-products, these are: wheat bran, wheat straw and alfalfa. Supplements are used to optimize the nutritional requirements of the mushrooms and enhance their fruiting. The various raw materials (basic ingredients and supplements) of the substrates should be mixed as homogeneously as possible and hydrated to a moisture content of 60-70 % depending on the species.

The substrate is packed in bags. The filled bags are placed in the autoclave for sterilization. Once sterilized, the bags are cooled to approximately 22 °C in the cooling room so that the initial inoculum or pure mycelium can be seeded.

The inoculated bags are transported to the incubation room. Here the trolleys are stacked with the bags and kept until the mycelium has invaded (colonized) the entire substrate. They must be perfectly air-conditioned in order to maintain the environmental conditions of the substrate (temperature, humidity, $CO_2$) within the optimum growth range of the species to be cultivated. From the incubation rooms, the substrate is transferred to the cultivation rooms, where the bags are opened and prepared for cultivation and subsequent harvesting of the mushrooms.

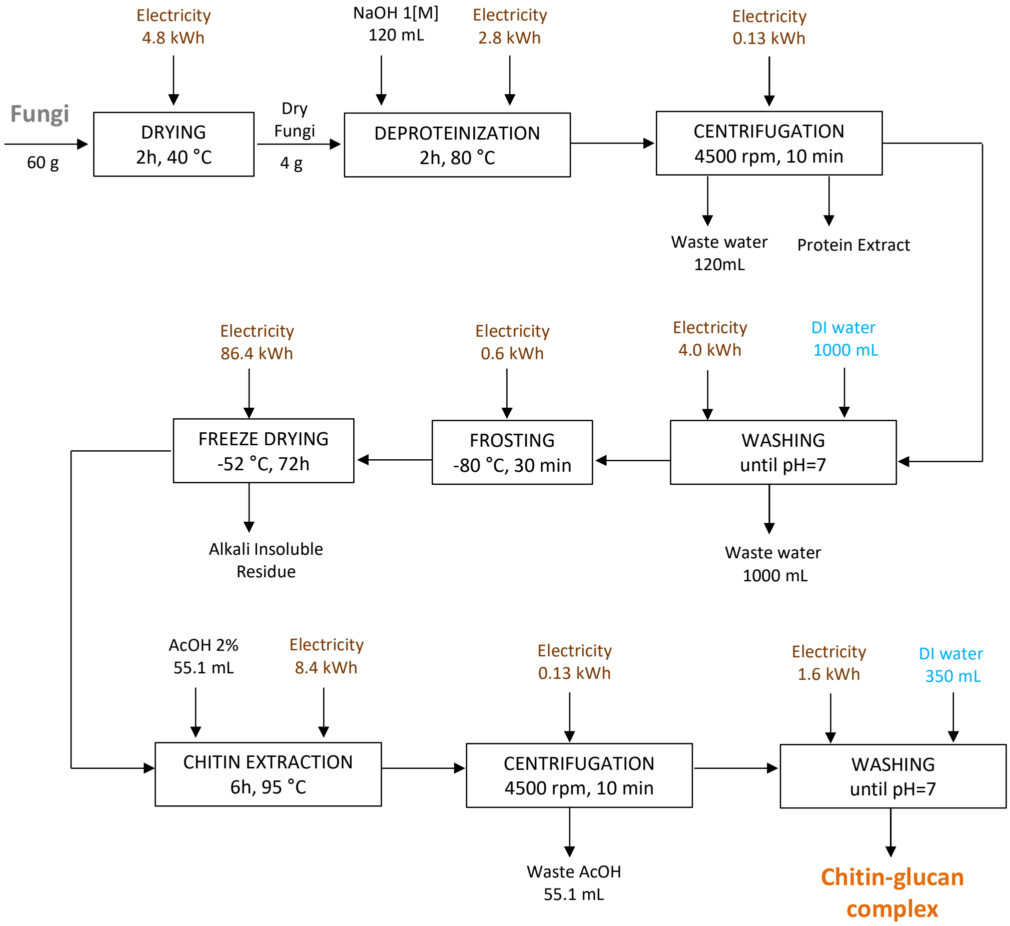


**Figure S1.** Process flowchart showing material and energy input. Note the energy consumption accounts for the whole equipment, which has been normalized to the amount of material that could be treated at each step.

**Table S1.** Modeled energy mix according to the Spanish mix for November 2023. Renewable sources are highlighted in green.

| **Source** | **Share** |
|---|---|
| *Wind* | 33.9 % |
| *Hydro* | 17.4 % |
| *Solar PV* | 9.4 % |
| *Solar thermal* | 0.6 % |
| *Other renewables* | 1.2 % |
| *Renewable residues* | 0.3 % |
| *Hydro pumping* | 1.7 % |
| *Nuclear* | 18.4 % |
| *Combined cycle, natural gas* | 10.6 % |
| *Coal* | 1.1 % |
| *Cogeneration* | 5.0 % |
| *Other non-renewables* | 0.5 % |

**Table S2.** Relative share to the global warming potential, acidification, eutrophication, and water use of energy, water, and materials use. Note that the total impacts may not be exactly 100.0% due to the rounding to obtain one significant decimal.

| Species | Global warming potential | | | Terrestrial acidification | | | Freshwater eutrophication | | | AWARE | | |
|---|---|---|---|---|---|---|---|---|---|---|---|---|
| | Energy share (%) | Water share (%) | Materials share (%) | Energy share (%) | Water share (%) | Materials share (%) | Energy share (%) | Water share (%) | Materials share (%) | Energy share (%) | Water share (%) | Materials share (%) |
| *AA* | 78.3 | 7.5 | 14.2 | 74.3 | 8.6 | 17.2 | 16.8 | 73.3 | 9.9 | 54.0 | 35.0 | 11.0 |
| *AAj* | 78.1 | 7.5 | 14.4 | 74.0 | 8.6 | 17.4 | 16.7 | 73.4 | 10.0 | 53.8 | 35.1 | 11.2 |
| *AB* | 79.3 | 7.3 | 13.4 | 75.4 | 8.4 | 16.2 | 17.4 | 73.2 | 9.5 | 55.3 | 34.6 | 10.2 |
| *AB-C* | 78.7 | 7.4 | 13.9 | 74.7 | 8.5 | 16.8 | 17.0 | 73.3 | 9.7 | 54.5 | 34.8 | 10.7 |
| *ABp* | 79.0 | 7.4 | 13.6 | 75.1 | 8.5 | 16.5 | 17.2 | 73.2 | 9.6 | 54.9 | 34.7 | 10.4 |
| *ABp-C* | 78.7 | 7.4 | 13.9 | 74.7 | 8.5 | 16.8 | 17.0 | 73.3 | 9.7 | 54.5 | 34.8 | 10.7 |
| *ABp-P* | 78.8 | 7.4 | 13.8 | 74.8 | 8.5 | 16.7 | 17.1 | 73.3 | 9.7 | 54.6 | 34.8 | 10.6 |
| *ABz* | 79.1 | 7.3 | 13.5 | 75.2 | 8.5 | 16.4 | 17.2 | 73.2 | 9.6 | 55.0 | 34.6 | 10.4 |
| *CI* | 79.3 | 7.3 | 13.4 | 75.4 | 8.4 | 16.2 | 17.3 | 73.2 | 9.5 | 55.2 | 34.6 | 10.2 |
| *Fva* | 78.4 | 7.4 | 14.1 | 74.4 | 8.6 | 17.0 | 16.9 | 73.3 | 9.8 | 54.2 | 34.9 | 10.9 |
| *FVb* | 78.9 | 7.4 | 13.8 | 74.9 | 8.5 | 16.6 | 17.1 | 73.3 | 9.7 | 54.7 | 34.8 | 10.6 |
| *GAN* | 76.7 | 7.7 | 15.6 | 72.5 | 8.9 | 18.7 | 15.9 | 73.5 | 10.5 | 52.1 | 35.6 | 12.3 |
| *GF* | 78.8 | 7.4 | 13.8 | 74.8 | 8.5 | 16.7 | 17.1 | 73.3 | 9.7 | 54.7 | 34.8 | 10.6 |
| *HC* | 78.7 | 7.4 | 13.9 | 74.7 | 8.5 | 16.8 | 17.0 | 73.3 | 9.7 | 54.5 | 34.8 | 10.7 |
| *HE* | 78.7 | 7.4 | 13.9 | 74.7 | 8.5 | 16.8 | 17.0 | 73.3 | 9.7 | 54.5 | 34.8 | 10.7 |
| *HTb* | 79.6 | 7.3 | 13.2 | 75.6 | 8.4 | 16.0 | 17.5 | 73.2 | 9.4 | 55.5 | 34.5 | 10.0 |
| *HTg* | 79.2 | 7.3 | 13.5 | 75.3 | 8.4 | 16.3 | 17.3 | 73.2 | 9.5 | 55.1 | 34.6 | 10.3 |
| *LE* | 77.8 | 7.5 | 14.7 | 73.7 | 8.7 | 17.6 | 16.5 | 73.4 | 10.1 | 53.4 | 35.2 | 11.4 |
| *PC* | 79.0 | 7.4 | 13.7 | 75.0 | 8.5 | 16.5 | 17.1 | 73.2 | 9.6 | 54.8 | 34.7 | 10.5 |
| *PE* | 79.1 | 7.3 | 13.6 | 75.1 | 8.5 | 16.4 | 17.2 | 73.2 | 9.6 | 55.0 | 34.7 | 10.4 |
| *PF* | 79.0 | 7.4 | 13.6 | 75.1 | 8.5 | 16.5 | 17.2 | 73.2 | 9.6 | 54.9 | 34.7 | 10.4 |
| *PN* | 79.2 | 7.3 | 13.5 | 75.2 | 8.5 | 16.4 | 17.3 | 73.2 | 9.5 | 55.1 | 34.6 | 10.3 |
| *PO* | 79.4 | 7.3 | 13.4 | 75.4 | 8.4 | 16.2 | 17.4 | 73.2 | 9.5 | 55.3 | 34.6 | 10.2 |
| *PS* | 78.9 | 7.4 | 13.7 | 74.9 | 8.5 | 16.6 | 17.1 | 73.3 | 9.7 | 54.7 | 34.7 | 10.6 |
| *SC* | 78.8 | 7.4 | 13.8 | 74.8 | 8.5 | 16.7 | 17.1 | 73.3 | 9.7 | 54.7 | 34.8 | 10.6 |

**Table S3.** The estimated crystalline structure parameters of isolated material. The samples containing glucans are shaded in brown.

| **Fungi species** | **Crystallinity[a] (%)** | **$L_{020}$ [a] (nm)** | **Fungi species** | **Crystallinity[a] (%)** | **$L_{020}$ [a] (nm)** |
|---|---|---|---|---|---|
| *AA* | 47.0 | 4.5 | *HC* | 55.0 | 4.6 |
| *AAj* | 28.0 | 2.3 | *HE* | 51.0 | 3.9 |
| *AB* | 55.0 | 4.5 | *HTb* | 55.0 | 3.9 |
| *ABc* | 53.0 | 4.7 | *HTg* | 46.0 | 3.9 |
| *ABp* | 67.0 | 3.9 | *LE* | 41.0 | 4.7 |
| *ABp-c* | 39.0 | 4.6 | *PC* | 47.0 | 5.4 |
| *ABz* | 52.0 | 4.4 | *PE* | 39.0 | 5.4 |
| *CI* | 42.0 | 4.4 | *PF* | 32.0 | 5.4 |
| *FVa* | 59.0 | 3.2 | *PN* | 67.0 | 3.3 |
| *FVb* | 62.0 | 4.1 | *PO* | 57.0 | 3.3 |
| *GAN* | 44.0 | 4.2 | *PS* | 57.0 | 3.5 |
| *GF* | 64.0 | 5.4 | *SC* | 78.0 | 4.3 |

[a]: values have been estimated using the same procedure to enable comparison.

**Table S4.** Characteristic FTIR bands ($cm^{-1}$) for the isolated chitin. The samples containing glucans are shaded in brown.

| Species | –OH stretching | –NH stretching | –CH groups | Amide I | Amide II | Amide III | C-N stretching | CH bending, symmetric $CH_3$ deformation | C-OH stretching | -CH deformation |
|---|---|---|---|---|---|---|---|---|---|---|
| *Chitin from shrinp* | 3444.7 | 3254.8 | 3101.0 | 1651.7 / 1621.3 | 1553.4 | 1308.9 | 1422.2 | 1375.0 | 1011.9 | 897.2 |
| *AA* | 3435.6 | 3252.4 | 2880.2 | 1636.3 | 1564.0 | 1312.3 | 1415.5 | 1371.1 | 1037.5 | 893.8 |
| *AAj* | 3333.4 | 3299.6 | 2911.0 | 1639.2 | 1562.1 | 1314.2 | 1423.2 | 1370.2 | 1032.7 | 891.0 |
| *AB-c* | 3433.6 | 3295.8 | 2886.0 | 1640.2 | 1556.3 | 1311.4 | 1414.5 | 1375.0 | 1031.7 | 898.7 |
| *ABp* | 34144 | 3272.6 | 2878.2 | 1638.2 | 1554.3 | 1313.3 | 1420.3 | 1375.0 | 1032.7 | 901.6 |
| *ABp-c* | 3390.2 | 3266.8 | 2886.0 | 1632.5 | 1555.3 | 1314.3 | 1417.4 | 1375.0 | 1033.7 | 898.7 |
| *ABz* | 3371.0 | 3288.0 | 2921.6 | 1635.3 | 1557.2 | 1314.3 | 1419.4 | 1376.0 | 1040.4 | 883.2 |
| *CI* | 3390.2 | 3270.7 | 2920.7 | 1632.5 | 1557.2 | 1314.3 | 1424.2 | 1374.0 | 1030.8 | 897.7 |
| *FVa* | 3428.8 | 3292.9 | 2919.7 | 1630.5 | 1559.2 | 1315.2 | 1423.2 | 1372.1 | 1036.6 | 892.9 |
| *FVb* | 3414.4 | 3274.5 | 2915.8 | 1633.4 | 1557.2 | 1313.3 | 1423.2 | 1374.0 | 1033.7 | 891.0 |
| *GAN* | 3368.1 | 3272.6 | 2890.8 | 1639.2 | 1554.3 | 1316.2 | 1416.5 | 1370.2 | 1045.2 | 893.8 |
| *GF* | 3422.1 | 3291.9 | 2887.9 | 1632.5 | 1552.4 | 1317.2 | 1422.2 | 1369.2 | 1037.5 | 887.1 |
| *HC* | 3410.5 | 3281.3 | 2922.6 | 1630.5 | 1556.3 | 1311.4 | 1423.2 | 1374.0 | 1031.7 | 890.9 |
| *HE* | 3418.2 | 3274.5 | 2921.6 | 1631.5 | 1554.3 | 1313.3 | 1422.2 | 1375.0 | 1030.8 | 894.8 |
| *HTb* | 3410.5 | 3281.3. | 2888.8 | 1630.5 | 1556.3 | 1313.3 | 1420.3 | 1374.0 | 1032.7 | 893.8 |
| *HTg* | 3406.6 | 3254.3 | 2900.4 | 1628.6 | 1558.2 | 1312.3 | 1414.5 | 1373.1 | 1035.6 | 897.7 |
| *LE* | 3361.3 | 3281.3 | 2891.7 | 1633.4 | 1559.2 | 1312.3 | 1421.3 | 1372.1 | 1034.6 | 893.8 |
| *PC* | 3402.8 | 3273.6 | 2912.0 | 1636.3 | 1555.3 | 1314.3 | 1424.2 | 1373.1 | 1034.6 | 892.9 |
| *PE* | 3393.1 | 3314.1 | 2924.5 | 1633.4 | 1558.2 | 1309.4 | 1416.5 | 1372.1 | 1034.6 | 890.9 |
| *PF* | 3432.7 | 3274.5 | 2924.5 | 1631.5 | 1559.2 | 1312.3 | 1421.3 | 1373.1 | 1034.6 | 891.0 |
| *PN* | 3434.6 | 3298.6 | 2921.6 | 1635.3 | 1558.2 | 1312.3 | 1420.3 | 1373.1 | 1033.7 | 893.8 |
| *PO* | 3419.2 | 3290.0 | 2892.7 | 1632.5 | 1554.3 | 1312.3 | 1419.3 | 1371.1 | 1035.6 | 893.8 |
| *PS* | 3442.3 | 3273.6 | 2884.0 | 1639.2 | 1559.2 | 1309.4 | 1421.3 | 1371.1 | 1034.6 | 894.8 |
| *SC* | 3412.4 | 3302.5 | 2887.9 | 1640.2 | 1547.6 | 1322.0 | 1423.2 | 1366.3 | 1027.9 | 900.6 |

**Table S5.** Degree of *N*-acetylation of isolated material. The samples containing glucans are shaded in brown.

| Fungi species | *N*-acetylation (%) | Fungi species | *N*-acetylation (%) |
|---|---|---|---|
| *Chitin from shrinp* | | | 99.8 |
| ***AA*** | 70.1 | ***HC*** | 95.5 |
| ***AAj*** | 59.6 | ***HE*** | 98.4 |
| ***AB*** | 65.6 | ***HTb*** | 78.4 |
| ***AB-c*** | 90.3 | ***HTg*** | 72.3 |
| ***ABp*** | 93.9 | ***LE*** | 58.2 |
| ***ABp-c*** | 98.7 | ***PC*** | 72.4 |
| ***ABz*** | 71.4 | ***PE*** | 65.5 |
| ***CI*** | 82.0 | ***PF*** | 73.1 |
| ***FVa*** | 59.7 | ***PN*** | 74.3 |
| ***FVb*** | 70.5 | ***PO*** | 56.7 |
| ***GAN*** | 57.9 | ***PS*** | 65.2 |
| ***GF*** | 53.0 | ***SC*** | 77.8 |

**Table S6.** Characteristic thermodegradation temperatures ($T_{10\%}$ and $T_{peak}$) and maximum degradation rates ($\alpha_{max}$) of freeze-dried chitin powder from fungi. The samples containing glucans are shaded in brown.

| Sample | $T_{10\%}$ (°C) | $T_{peak}$ (°C) | $\alpha_{max}$ (%/°C) |
|---|---|---|---|
| *Chitin from shrinp* | 313.5 | 396.5 | 1.55 |
| *AA* | 260.7 | 321.7 | 1.17 |
| *AAj* | 270.3 | 320.6 | 1.24 |
| *AB* | 254.0 | 312.1 | 0.82 |
| *AB-c* | 260.7 | 321.1 | 0.91 |
| *ABp-c* | 270.2 | 325.5 | 1.05 |
| *ABz* | 260.4 | 314.2 | 1.10 |
| *CI* | 260.9 | 315.2 | 1.26 |
| *FVa* | 279.0 | 324.5 | 1.74 |
| *FVb* | 270.1 | 321.2 | 1.42 |
| *GAN* | 256.8 | 328.1 | 1.19 |
| *GF* | 265.7 | 323.8 | 1.11 |
| *HC* | 246.4 | 313.2 | 0.81 |
| *HE* | 257.4 | 325.8 | 0.70 |
| *HTb* | 256.5 | 322.1 | 0.85 |
| *HTg* | 244.3 | 310.1 | 0.89 |
| *LE* | 267.6 | 315.0 | 1.50 |
| *PC* | 260.0 | 320.7 | 1.38 |
| *PE* | 272.2 | 321.8 | 1.45 |
| ***PF*** | 258.3 | 313.1 | 1.18 |
| *PN* | 266.3 | 325.7 | 1.23 |
| *PO* | 263.1 | 319.4 | 1.13 |
| *PS* | 260.2 | 324.5 | 0.95 |
| *SC* | 268.9 | 315.0 | 1.26 |

**AA**

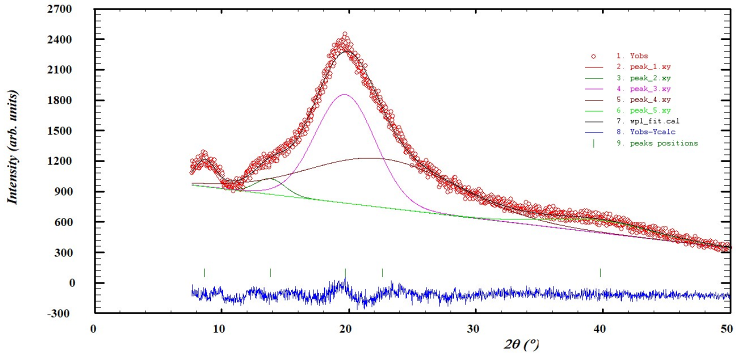
Intensity (arb. units)
2θ (°)
1. Yobs
2. peak_1.xy
3. peak_2.xy
4. peak_3.xy
5. peak_4.xy
6. peak_5.xy
7. wpl_fit.cal
8. Yobs-Ycalc
9. peaks positions

**AAj**

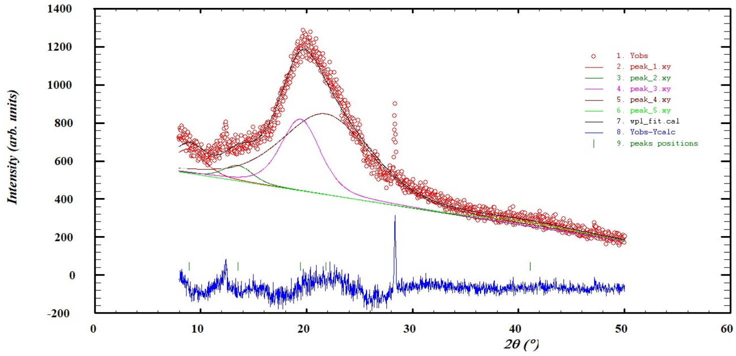
Intensity (arb. units)
2θ (°)
1. Yobs
2. peak_1.xy
3. peak_2.xy
4. peak_3.xy
5. peak_4.xy
6. peak_5.xy
7. wpl_fit.cal
8. Yobs-Ycalc
9. peaks positions

**AB**

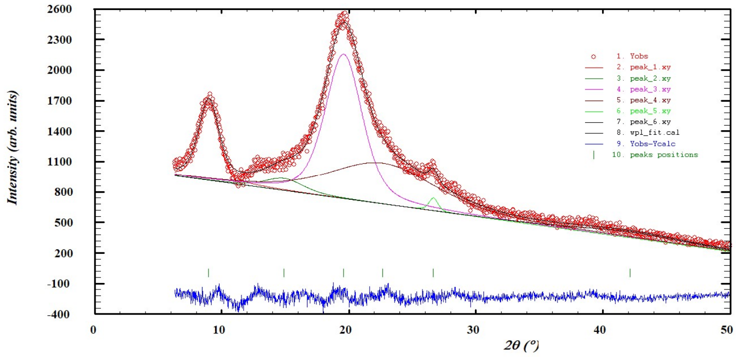
Intensity (arb. units)
2θ (°)
1. Yobs
2. peak_1.xy
3. peak_2.xy
4. peak_3.xy
5. peak_4.xy
6. peak_5.xy
7. peak_6.xy
8. wpl_fit.cal
9. Yobs-Ycalc
10. peaks positions

**AB-c**

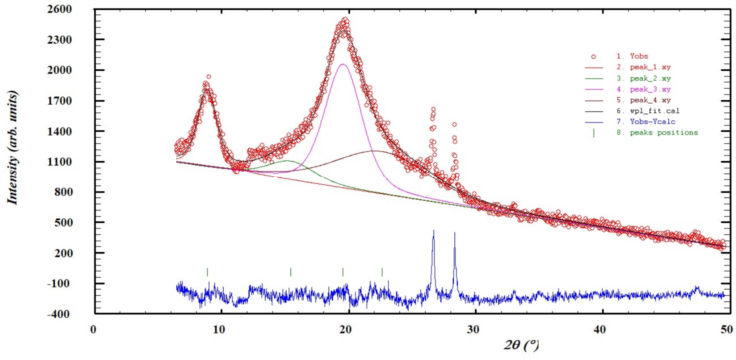
Intensity (arb. units)
2θ (°)
1. Yobs
2. peak_1.xy
3. peak_2.xy
4. peak_3.xy
5. peak_4.xy
6. wpl_fit.cal
7. Yobs-Ycalc
8. peaks positions

## ABp

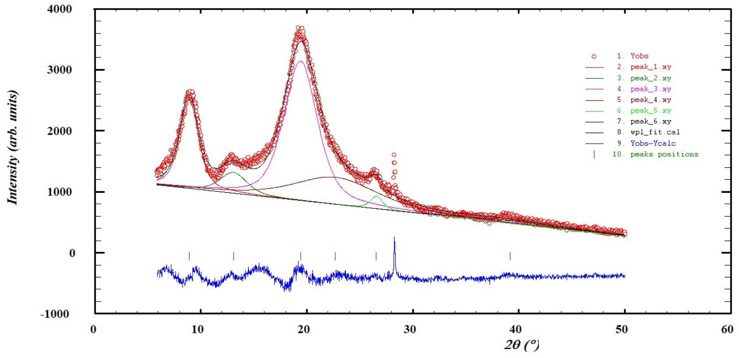


## ABp-c

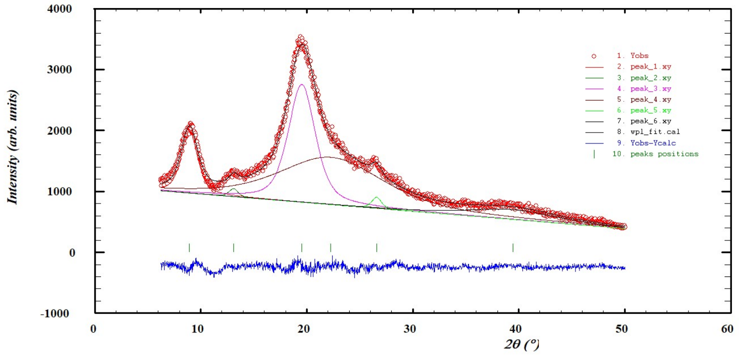


## ABz

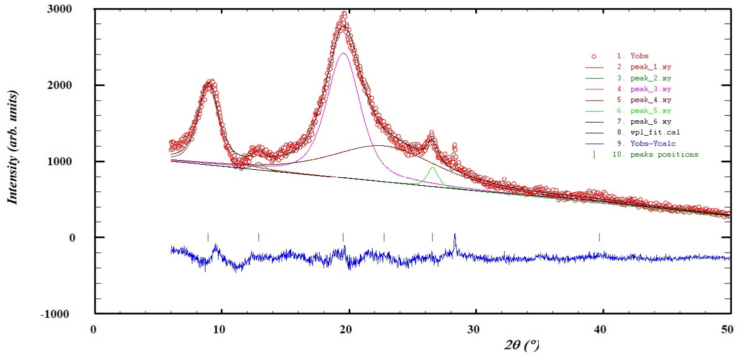


## CI

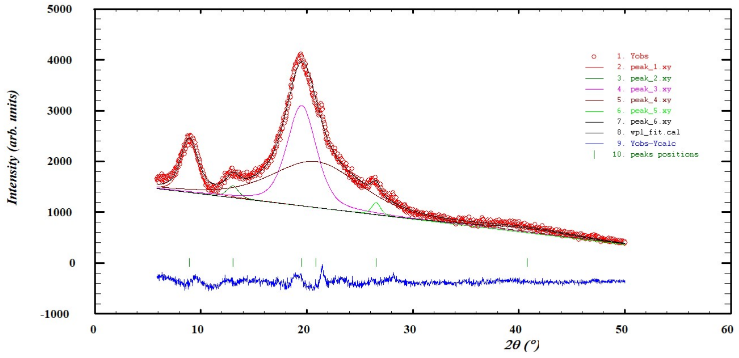

## FVa

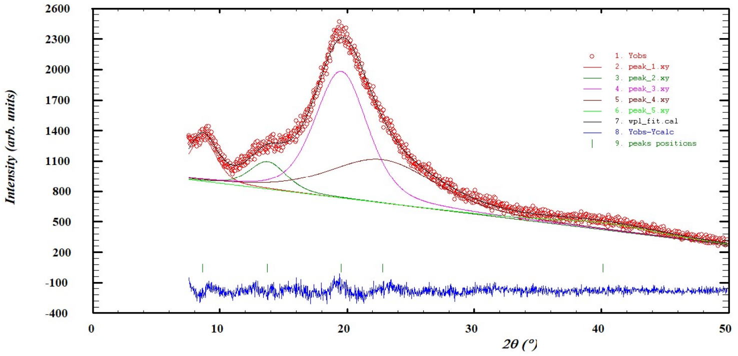


## FVb

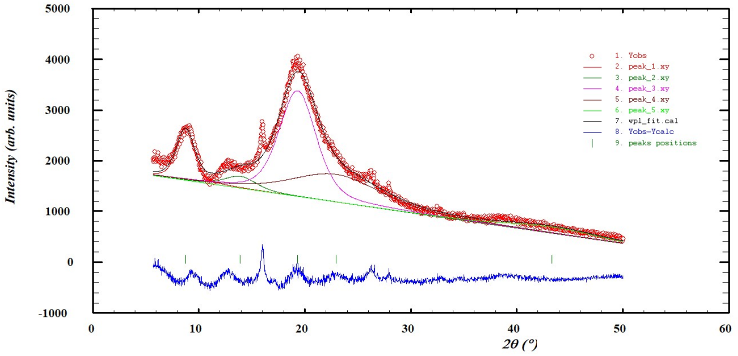


## GAN

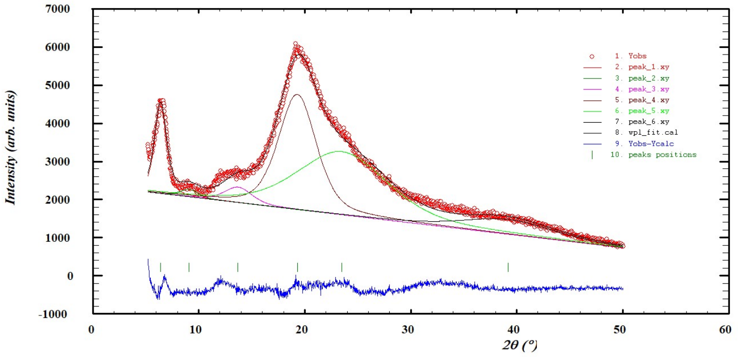


## GF

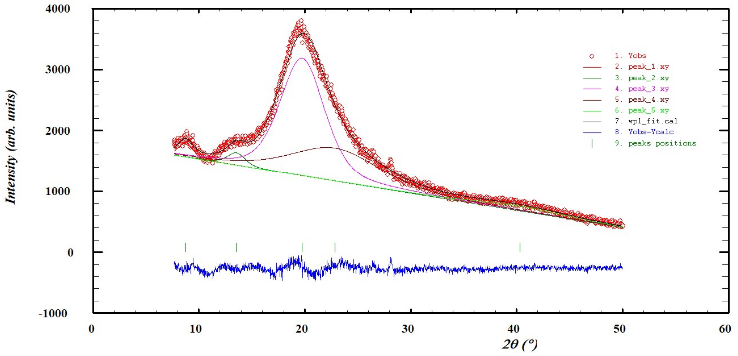

# HC

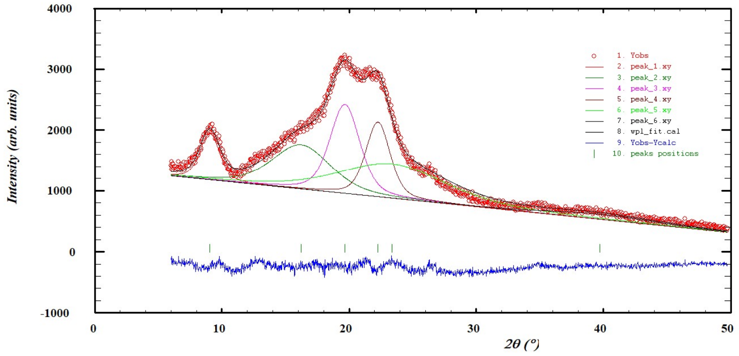
Intensity (arb. units)
2θ (°)
1. Yobs
2. peak_1.xy
3. peak_2.xy
4. peak_3.xy
5. peak_4.xy
6. peak_5.xy
7. peak_6.xy
8. wpl_fit.cal
9. Yobs-Ycalc
10. peaks positions

# HE

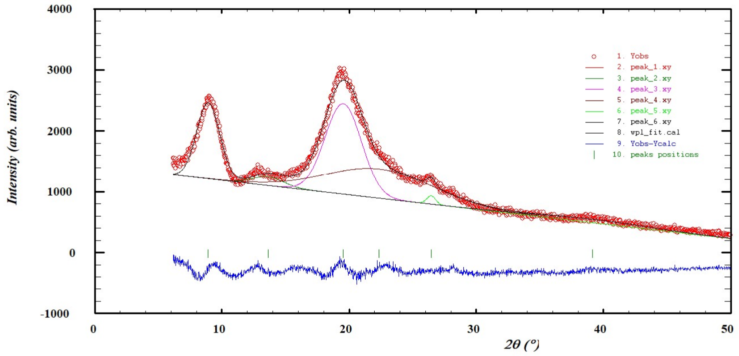
Intensity (arb. units)
2θ (°)
1. Yobs
2. peak_1.xy
3. peak_2.xy
4. peak_3.xy
5. peak_4.xy
6. peak_5.xy
7. peak_6.xy
8. wpl_fit.cal
9. Yobs-Ycalc
10. peaks positions

# HTb

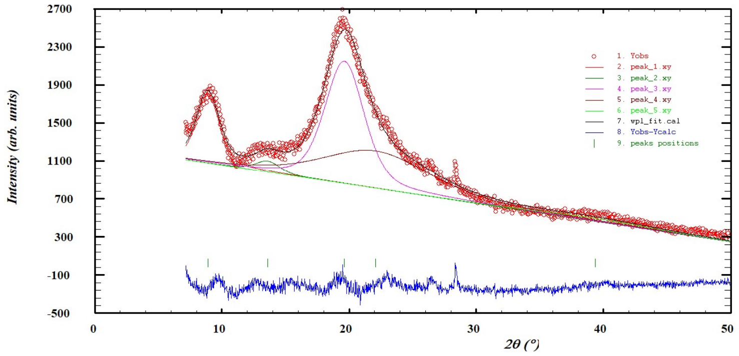
Intensity (arb. units)
2θ (°)
1. Yobs
2. peak_1.xy
3. peak_2.xy
4. peak_3.xy
5. peak_4.xy
6. peak_5.xy
7. wpl_fit.cal
8. Yobs-Ycalc
9. peaks positions

# HTg

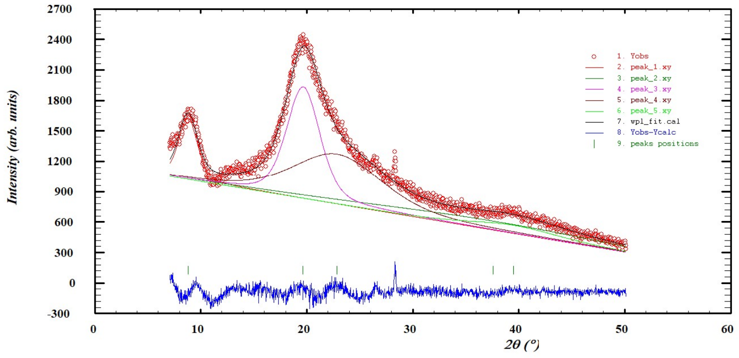
Intensity (arb. units)
2θ (°)
1. Yobs
2. peak_1.xy
3. peak_2.xy
4. peak_3.xy
5. peak_4.xy
6. peak_5.xy
7. wpl_fit.cal
8. Yobs-Ycalc
9. peaks positions

# LE

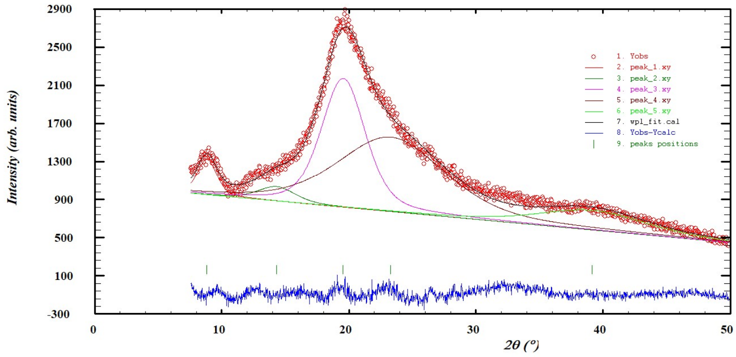

Intensity (arb. units)
2θ (°)
1. Yobs
2. peak_1.xy
3. peak_2.xy
4. peak_3.xy
5. peak_4.xy
6. peak_5.xy
7. wpl_fit.cal
8. Yobs-Ycalc
9. peaks positions


# PC

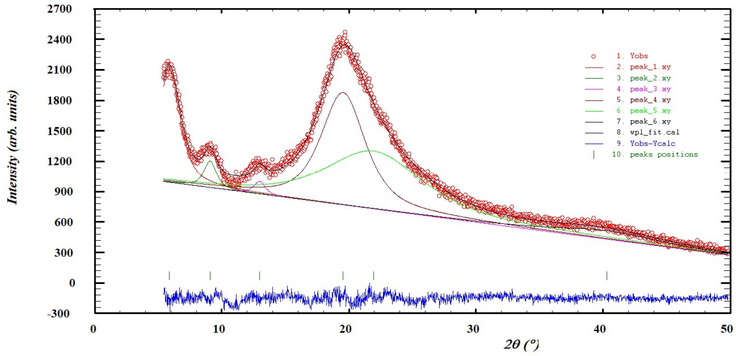

Intensity (arb. units)
2θ (°)
1. Yobs
2. peak_1.xy
3. peak_2.xy
4. peak_3.xy
5. peak_4.xy
6. peak_5.xy
7. peak_6.xy
8. wpl_fit.cal
9. Yobs-Ycalc
10. peaks positions


# PE

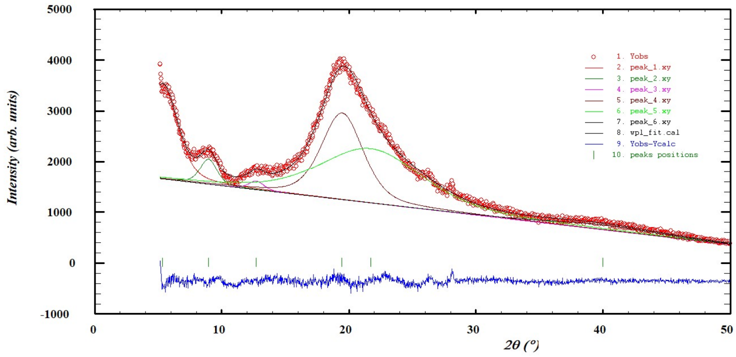

Intensity (arb. units)
2θ (°)
1. Yobs
2. peak_1.xy
3. peak_2.xy
4. peak_3.xy
5. peak_4.xy
6. peak_5.xy
7. peak_6.xy
8. wpl_fit.cal
9. Yobs-Ycalc
10. peaks positions


# PF

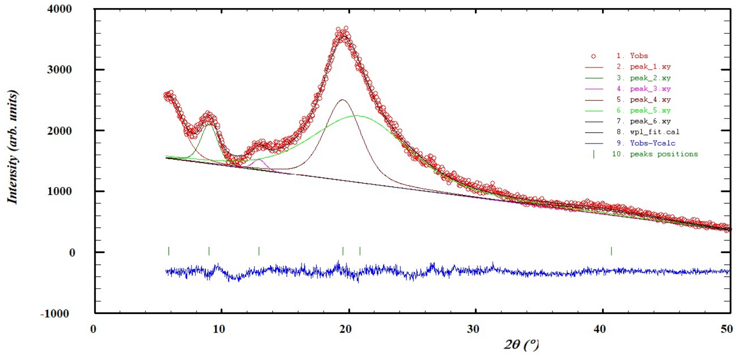

Intensity (arb. units)
2θ (°)
1. Yobs
2. peak_1.xy
3. peak_2.xy
4. peak_3.xy
5. peak_4.xy
6. peak_5.xy
7. peak_6.xy
8. wpl_fit.cal
9. Yobs-Ycalc
10. peaks positions

## PN

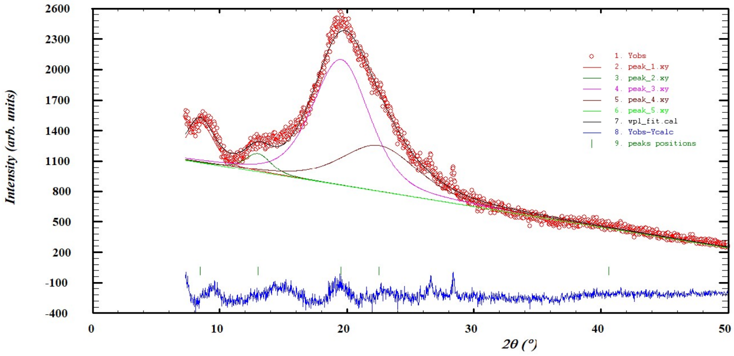


## PO

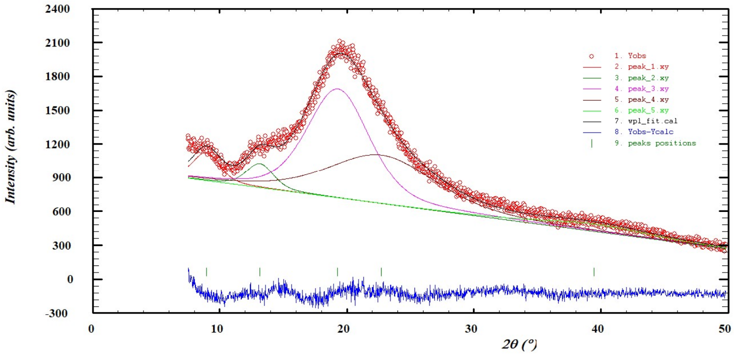


## PS

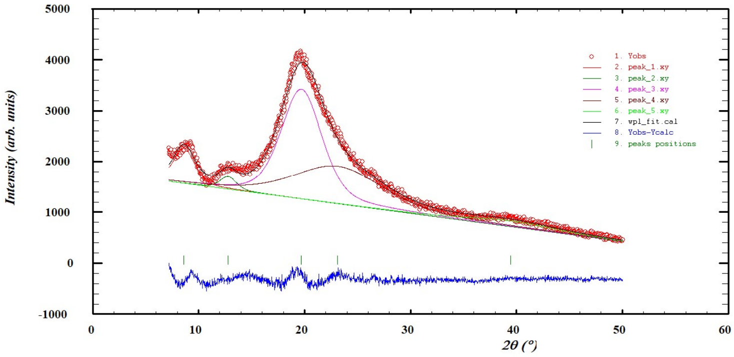


## SC

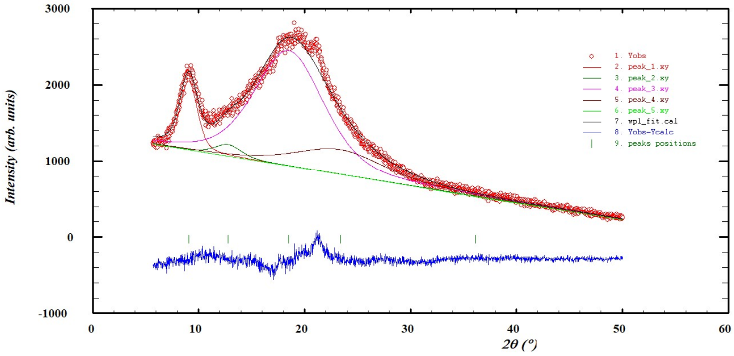


**Figure S2.** Deconvoluted XRD patterns of chitin isolated from 22 different fungal species. Vertical sticks indicate peak positions.

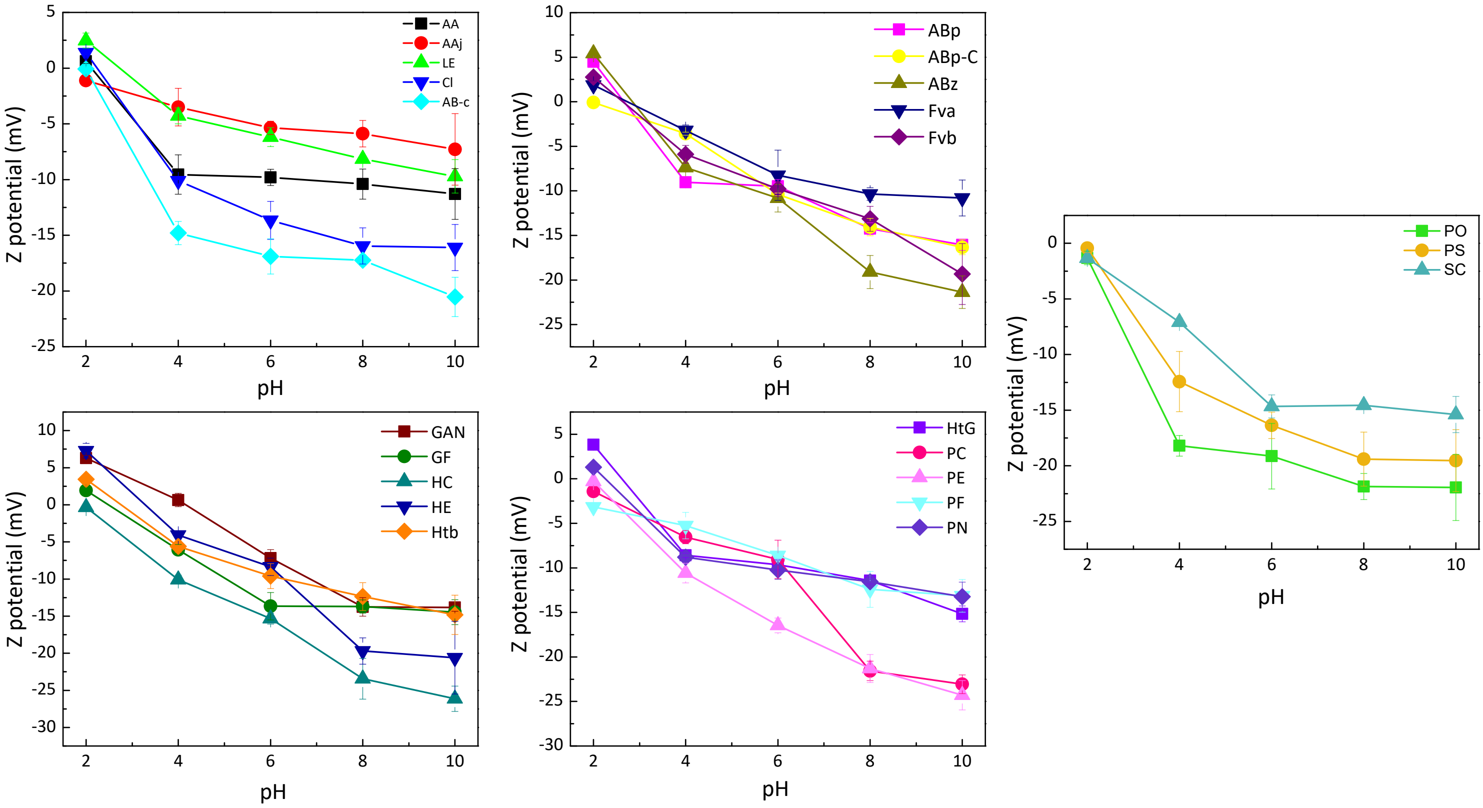


**Figure S3.** $\zeta$ -potential of isolated material at varying pH.
